# Molecular Epidemiology of *Human Parainfluenza Virus Type 3* in Children With Acute Respiratory Tract Infection in Hangzhou

**DOI:** 10.1111/irv.13351

**Published:** 2024-07-04

**Authors:** Ya‐jun Guo, Lin Li, Qin‐rui Lai, Ying‐shuo Wang, Wei Li

**Affiliations:** ^1^ Department of Clinical Laboratory, The Children's Hospital Zhejiang University School of Medicine Hangzhou China; ^2^ Department of Infectious Diseases, Fujian Children's Hospital (Fujian Branch of Shanghai Children's Medical Center), National Regional Medical Center, College of Clinical Medicine for Obstetrics & Gynecology and Pediatrics Fujian Medical University Fuzhou China; ^3^ Department of Respiratory, The Children's Hospital Zhejiang University School of Medicine Hangzhou China

**Keywords:** acute respiratory tract infection, children, COVID‐19, *human parainfluenza virus type 3*, L gene

## Abstract

**Background:**

Since the outbreak of COVID‐19, China has undertaken a variety of preventative and control measures, effectively reducing the incidence of numerous infectious diseases among the pediatric population in Hangzhou. We aim to investigate the genetic and epidemiological characteristics of *Human parainfluenza virus*‐3 (HPIV‐3) in pediatric patients during this period.

**Methods:**

A total of 1442 pharyngeal swab samples were collected from outpatients and inpatients with a diagnosis of acute respiratory tract infections (ARTIs) from November 2020 to March 2021. HPIV‐3 was detected by quantitative real time polymerase chain reaction (qRT‐PCR). The L gene of HPIV‐3 positive samples was amplified and sequenced.

**Results:**

Among 1442 children with ARTI, the positive rate of HPIV‐3 was 7.07% (102/1442). The positive detection rate was the highest in the 6‐month to 1‐year age group. Coinfection was observed in 36 HPIV‐3‐positive samples (35.29%, 36/102), and *adenovirus* (ADV) was the most common coinfecting virus (63.89%, 23/36). The L gene of 48 HPIV‐3 positive samples was sequenced. The nucleotide sequence analysis showed high consistency (92.10%–99.40%), and all strains belonged to C3a.

**Conclusions:**

During study periods, the positive detection rate of HPIV‐3 among children is high, and the highest proportion of coinfection was observed in HPIV‐3 mixed ADV infection. Phylogenetic analysis revealed that the nucleotide sequence of the L gene of HPIV‐3 was highly consistent, and the main epidemic strain in this area was the C3a subtype.

## 1 Introduction

*Human parainfluenza virus* (HPIV) belongs to Paramyxoviridae family [1], which is a nonsegmented negative‐strand RNA virus. Seasonal HPIV epidemics result in a significant burden of disease in children and account for 40.00% of pediatric hospitalizations for lower respiratory tract illnesses (LRTIs) and 75.00% of croup cases [2]. *Parainfluenza viruses* are associated with a wide spectrum of illnesses which include otitis media, pharyngitis, conjunctivitis, croup, tracheobronchitis, and pneumonia. Uncommon respiratory manifestations include apnea, bradycardia, parotitis, and respiratory distress syndrome and rarely disseminated infection.

According to the genetic and serological characteristics, HPIV can be divided into four serotypes: HPIV‐1, HPIV‐2, HPIV‐3, and HPIV‐4 [3]. HPIV‐1 and HPIV‐3 are the most common causes of upper and lower respiratory tract infections, whereas infections with HPIV‐2 and HPIV‐4 are comparatively rare [4, 5, 6, 7]. HPIV‐3‐induced infections manifest typical symptoms of epidemic respiratory diseases, which are easily confused with other respiratory viral infections [8]. The genome of HPIV‐3 contains 15,462 nucleotides, composed of 6 genes (nucleoprotein [NP]–phosphoprotein [P]–matrix [M]–fusion [F]–hemagglutinin‐neuraminidase [HN]–large [L]) that code for eight proteins (NP, P/D/C, M, F, HN, and L proteins) [9]. The largest protein in HPIV‐3, known as protein L, consists of about 6800 nucleotides and encodes 2333 amino acids. It carries out enzymatic activities during viral replication and transcription [10].

Following the outbreak of novel coronavirus disease (COVID‐19), China initiated many preventive strategies and control measures, including travel restrictions, social seclusion, and mask use. For the most part, children were guided to regularly wash their hands and wear masks [11]. According to recent studies, these efforts effectively lowered the frequency of many infectious diseases in children in Hangzhou, the capital of Zhejiang province in eastern China [12, 13]. However, from November 2020 to March 2021, the region witnessed a high infection rate of HPIV‐3. Therefore, our goal was to investigate the genetic and epidemiological traits of HPIV‐3 in pediatric patients throughout this time.

## 2 Methods

### 2.1 Sample Collection

One thousand four hundred forty‐two throat swabs were collected from patients with ARTI between November 2020 and March 2021 [14]. A 2.5 mL viral transport medium (KaiBiLi, Hangzhou, China) was used to preserve the throat swabs. The Children's Hospital Ethics Committee at Zhejiang University School of Medicine approved the study, and all study participants' parents or legal guardians provided written informed consent.

### 2.2 Detection of HPIV‐3

RNA was extracted from a 300 μL material using the paramagnetic particle method (Catalog Z‐ME‐0044, Shanghai Zhijiang Biotechnology Co., Ltd., China). An Applied Biosystems 7500 Real‐Time PCR System (Applied Biosystems, Foster City, CA, USA) was used to detect the HPIV‐3 using 5 μL extracted nucleic acids mixed with 20 μL PCR reagents (Shanghai Biogerm Medical Technology Limited Company, Shanghai, China). The thermocycling procedure was as follows: 15 min of reverse transcription at 50°C, 5 min at 95°C, 45 cycles at 95°C for 15 s, and 40 s at 55°C. The manufacturer's instructions were strictly followed during every step of the experiment. Real‐time RT‐PCR was also used to identify the *influenza* A and B viruses, the *human metapneumovirus* (HMPV), the *respiratory syncytial virus* (RSV), the *adenovirus* (ADV), the HPIV‐1, and the HPIV‐2 as controls (Shanghai Biogerm Medical Technology Limited Company, Shanghai, China).

### 2.3 PCR Amplification of the L Protein Gene

The L gene from HPIV‐3 positive samples was amplified in two steps. First, using the MonScript RTIII Super Mix with dsDNase (Monad Biotech Co., Ltd., China), the viral RNA that had been isolated was reverse‐transcribed into cDNA. Second, using 2Taq MasterMix (CWBio Co., Ltd., China) and the following parameters, the partial L gene was amplified: 2 min at 94°C, 35 cycles at 94°C for 30 s, 55°C for 30 s, and 72°C for 40 s. HPIV‐3‐F:5′‐TGAATATAACAGATGTAATTGGTCAACGAG‐3′ and HPIV‐3‐R:5′‐CCCGCCTAATTTATGTCTCTTGTCA‐3′ were the PCR primers utilized in this investigation. Finally, TSingKe Biological Technology Co., Ltd. (Hangzhou, China) received the PCR products for sequencing.

### 2.4 Sequence Alignment

The HPIV‐3 L gene sequences were evaluated and compared with the public HPIV‐3 L gene, using the basic local alignment search tool (BLAST) (found at http://www.ncbi.nlm.gov/BLAST/). Through DNAstar 7, several sequence alignments were carried out with ClustalW. The MEGA version 7.0 was used to perform phylogenetic analysis. The Kimura 2Parameter technique was used to determine genetic distances. The neighbor‐joining approach was used to create dendrograms, and 1000 repetitions were used in bootstrap analyses.

### 2.5 Statistical Analysis

All analyses were performed using IBM SPSS Statistics version 25.0 (IBM Corp., Armonk, NY, USA). In accordance with their skewed distribution, continuous variables were compared using a nonparametric statistical test and presented as the median value with an interquartile range. The chi‐square (*χ* ^2^) test was used to compare percentage descriptions of categorical variables. Two‐sided *p* values and 95.00% confidence intervals were presented throughout and *p* value < 0.05 represented statistically significant.

## 3 Results

**FIGURE 1:**
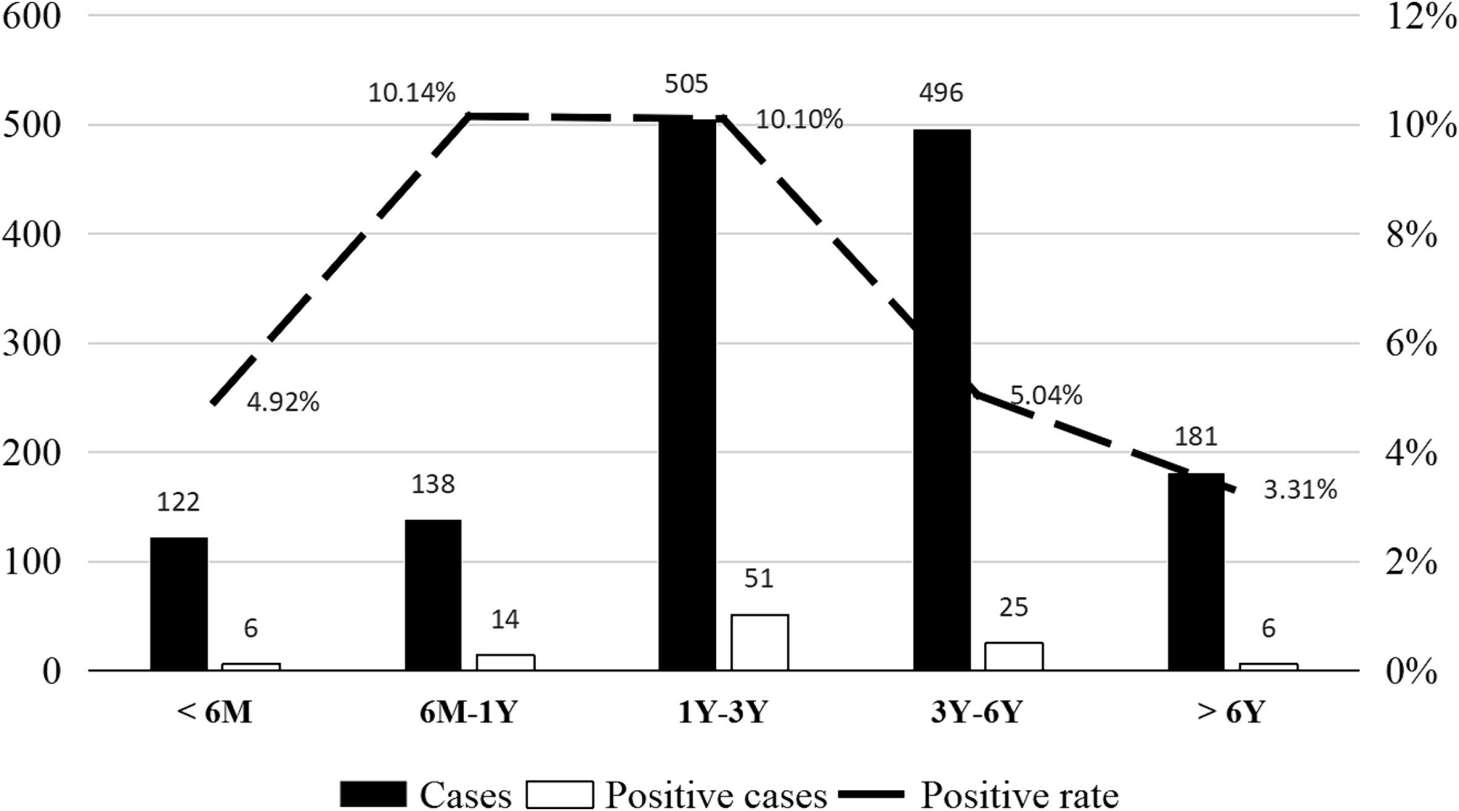
Age distribution of the positive rates in children with HPIV‐3 infection. M = month; Y = year.

A total of 1442 pediatric patients with ARTI were included in this study, comprising 801 males and 641 females. Among these, 102 cases tested positive for HPIV‐3, with 55 males (6.87%) and 47 females (7.33%) affected. The positive rates of HPIV‐1 and HPIV‐2 were 1.04% (15/1442) and 0.14% (2/1442). The gender distribution in HPIV‐3‐infected patients was not significantly different (*χ* ^2^ = 0.118, *p* = 0.732). As presented in Table 1, fever (82.35%, 84/102) and cough (81.37%, 83/102) were the primary symptoms observed in HPIV‐3‐infected patients, followed by coughing up phlegm (57.84%, 59/102), runny nose (32.35%, 33/102), and rales and phlegm sounds (23.53%, 24/102). The age range of infected children was 1 month to 12 years and 6 months. The positive detection rate for HPIV‐3 was highest in the 6‐month to 1‐year age group (10.15%, 14/138) and lowest in the > 6‐year age group (3.31%) with 6 out of 122 (4.92%, 6/122) cases in the 0‐ to 6‐month age group, 51 out of 505 (10.10%, 51/505) cases in the 1‐ to 3‐year age group, and 25 out of 496 cases (5.04%, 25/496) in the 3‐ to 6‐year age group, as illustrated in Figure 1. Table 2 displays the coinfectious cases observed in 36 out of 102 (35.29%, 36/102) HPIV‐3‐positive samples. The most commonly detected coinfecting virus was ADV (63.89%, 23/36), followed by RSV (30.56%, 11/36) and HMPV (5.56%, 2/36).

**TABLE 1:** Clinical characteristics of children with *human parainfluenza virus‐3* (*N* = 102).

Clinical characteristics	Number of HPIV‐positive children ***n*** (%)
Fever	84 (82.35%)
Cough	83 (81.37%)
Coughing up phlegm	59 (57.84%)
Runny nose	33 (32.35%)
Rales and phlegm sounds Shortness of breath	24 (23.53%) 8 (7.84%)
Wheezing	2 (1.96%)
Oxygen therapy Sore throat	2 (1.96%) 1 (0.98%)

**TABLE 2:** Coinfections of HPIV‐3 of other viruses (*N* = 36).

Virus composition	Number of cases (%)
HPIV‐3 + ADV	23 (63.89)
HPIV‐3 + RSV	11 (30.56)
HPIV‐3 + HMPV	2 (5.56)

**FIGURE 2:**
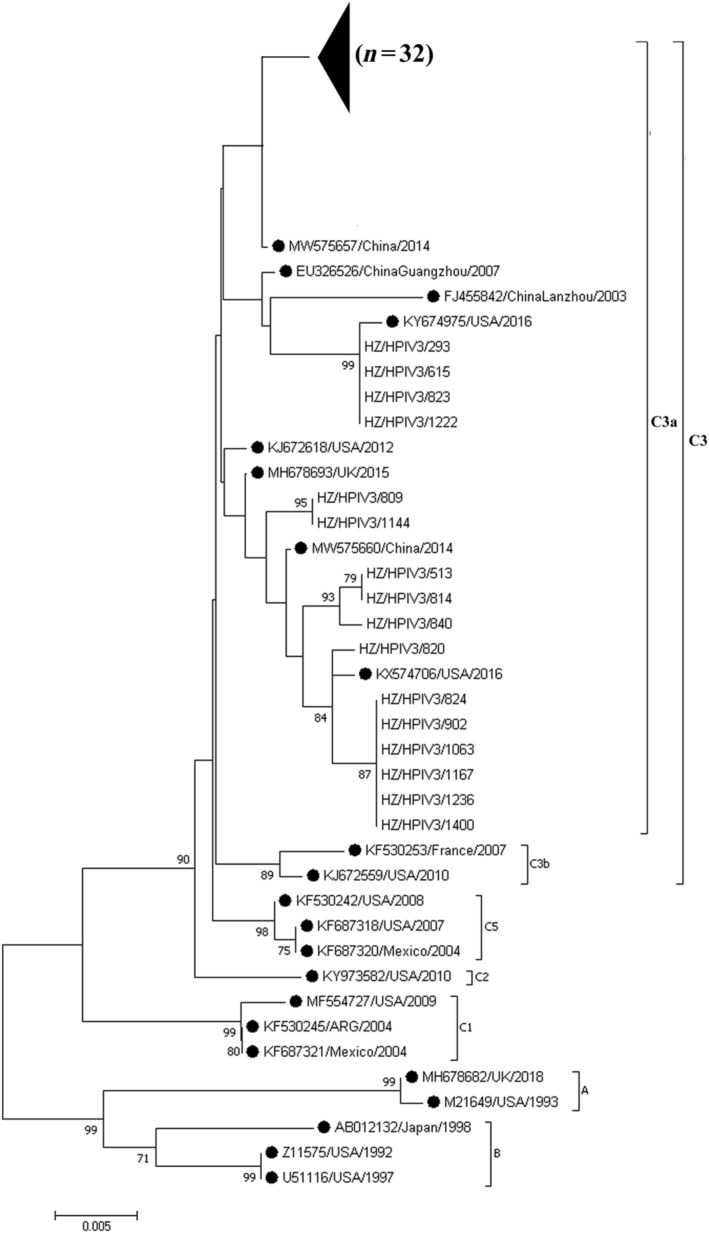
Phylogenetic analysis of the nucleotide sequences from the L genes of HPIV‐3. Evolutionary analysis was conducted using MEGA 7. The evolutionary history was inferred using the neighbor‐joining method, and 1000 repetitions were used in bootstrap analyses.

Forty‐eight L gene fragments of HPIV‐3 were successfully sequenced, with a length of approximately 727 bp. As shown in Table S1, the homology analysis revealed a high consistency of nucleotide sequence (92.10%–99.40%). Phylogenetic analysis of HPIV‐3 indicated that all the HPIV‐3 samples belonged to genotype C, subtype C3a. By comparing HPIV‐3 sequences obtained in this study with those registered in GenBank, we found that most of the strains (32 clinical strains) detected in Hangzhou exhibited almost identical nucleotide sequences to the strain from Beijing, China, in 2014 (MW575657/China/2014), with a percentage identity of 99.32% (Figure 2). Moreover, four strains were highly similar to the strain from the United States in 2016 (KY674975/USA/2016), two strains were most closely related to the strain from the United Kingdom in 2015 (MH678693/UK/2015), three strains showed marked similarity to the strain from Beijing, China, in 2014 (MW575660/China/2014), and seven strains were exhibited substantial resemblance to the strain from the United States in 2016 (KX574706/USA/2016).

## 4 Discussion

Among the four serotypes of HPIV, HPIV‐3 is the predominant type of HPIVs circulating in China, accounting for up to 90.00% of infections, and is known as an important cause of ARTI in children [15]. Among HPIV1‐3, HPIV‐3 accounts for the main proportion in our study (85.70%, 102/119). The prevalence of HPIV‐3 in children did not diminish following the COVID‐19 outbreak, as it did for other viruses. The positive detection rate in our study was 7.07%, which is far higher compared to that in the United States in 2011–2019 (2.51%) [16]. It also surpasses the detection rates of 4.30% and 4.00% reported respectively by two studies performed in Hangzhou (2001–2006) and Wenzhou (2008–2017), two major cities in Zhejiang province [17, 18]. This may be attributed to strict preventive measures taken after COVID‐19, which effectively controlled the spread of influenza A and B, but resulted in an increased prevalence of HPIV‐3. Our study found that the primary symptoms of HPIV‐3 infection were fever and cough (above 80.00%). The positivity rate for HPIV‐3 was the highest in children from 6 months to 3 years old, based on our findings: The positivity rates in the 6‐month to 1‐year age group and 1‐ to 3‐year age group were 10.15% and 10.10%, respectively. Differing from the previous study, we found that children under the age of 1 year had the highest HPIV‐3 positive rate [15]. This is further substantiated by additional research, suggesting that the probability of contracting HPIV‐3 is indeed highest in children under 1 year old [19, 20]. Several studies have reported coinfection of HPIV‐3 with other respiratory viruses [21, 22, 23]. In the present study, the rate of HPIV‐3 coinfection was 35.29%. The respiratory viruses coinfected with HPIV‐3 included the following: ADV, RSV, and HMPV.

The HPIV‐3 strains were classified into three clusters (A, B, and C), with cluster C being further subdivided into subclusters (C1–C5) and genetic lineages in C1 and C3 subclusters [24]. Numerous studies revealed that cluster C was the predominant strain throughout the world [25, 26], as C3c and C1b were predominant subtypes in Israel in 2012–2015 [3] and C3a was the major subtype in Croatia in 2011–2015 [9]. In this study, our findings showed that the subgenotype C3a was dominant, while other subgenotypes were not identified. The absence of additional subgenotypes could be attributed to our brief study time. Therefore, additional data from various countries, as well as long‐term observations, are necessarily required to track the epidemic characteristics of HPIV‐3 subgenotypes following COVID‐19.

The L protein is a part of the RNA‐dependent RNA polymerase complex that transcribes the genomic RNA encapsidated by N protein but not the naked RNA. The L protein appears to contain posttranscriptional modification activities [22]. In this study, we found that the conservation of L gene sequences between clinical strains was 92.10%–99.40% [27]. The high nucleotide acid identity levels of the L gene found in our study proved once again that the HPIV‐3 L protein is highly conserved. Moreover, sequences from our study were almost identical to the viruses isolated both in China and other regions (United Kingdom and United States) suggesting that traveling might affect the evolution of new epidemic viruses in distant places.

The primary limitation of this study was the incomplete sequencing of the L gene. Further research is necessary to validate the complete characteristics of the whole L gene. Additionally, our single‐center patient sample and inadequate study period may not accurately represent averages across China's diverse regions, highlighting the necessity for multicenter collaboration. We aim to collaborate with more centers in future studies.

## 5 Conclusions

In conclusion, this study was about the epidemiological and genetic characteristics of L gene of HPIV‐3 in pediatric patients in Hangzhou after the peak of COVID‐19. Despite the implementation of prevention and control measures in China, the prevalence of HPIV‐3 remains high, particularly among children aged 6 months to 1 year, among whom it not only accounts for the highest positive detection rates but ADV is also the most prevalent coinfecting virus. Phylogenetic analysis revealed that the C3a was the dominant strain from November 2020 to March 2021 in Hangzhou.

## Author Contributions

**Ya‐jun Guo:** conceptualization, data curation, formal analysis, investigation, writing–original draft. **Lin Li:** data curation, investigation, formal analysis. **Qin‐rui Lai:** project administration, data curation. **Ying‐shuo Wang:** investigation, supervision. **Wei Li:** conceptualization, supervision, writing–review and editing.

## Ethics Statement

The study was approved by the Children's Hospital Ethics Committee at Zhejiang University School of Medicine.

## Conflicts of Interest

The authors declare no conflicts of interest.

### Peer Review

The peer review history for this article is available at https://www.webofscience.com/api/gateway/wos/peer‐review/10.1111/irv.13351.

## Supporting information


**Table S1** The sequences from the L genes of HPIV‐3.

## Data Availability

The data that support the findings of this study are available from the corresponding authors, upon reasonable request.
