# Supplementary material for: Molecular Epidemiology of Human Parainfluenza Virus Type 3 in Children With Acute Respiratory Tract Infection in Hangzhou
Source: Influenza Other Respir Viruses. 2024 Jul 4;18(7):e13351. doi: 10.1111/irv.13351 (PMC11224502; doi:10.1111/irv.13351)
Supplement: Supplementary file 1 — Table S1 The sequences from the L genes of HPIV‐3. [file IRV-18-e13351-s001.docx]

>HZ/HPIV3/110

AACCCCCGATAGAGATAGTTGAAGTATTTATAGCAGATAGTAAATTTGATCCTCATCAGATATTGGATTATGTAGAATCTGGGGATTGGTTAGATGATCCAGAATTTAATATTTCTTATAGTCTCAAAGAAAAGGAGATCAAACAAGAAGGTAGACTCTTTGCAAAAATGACATACAAAATGAGAGCTACACAGGTTTTATCAGAGACACTACTTGCAAACAATATAGGAAAATTCTTTCAAGAAAATGGGATGGTGAAGGGAGAGATTGAATTACTTAAGAGATTAACGACTATATCAATATCAGGAGTTCCACGATATAATGAAGTGTACAATAATTCTAAAAGCCATACAGATGATCTTAAAACTTATAATAAAATAAGTAATCTTAATTTATCTTCTAATCAGAAATCAAAGAAATTTGAATTCAAGTCAACGGATATTTACAATGATGGATATGAGACTGTAAGCTGTTTCCTAACAACAGATCTCAAAAAATATTGTCTTAACTGGAGATATGAATCAACAGCTCTATTTGGAGAAACTTGCAATCAAATATTTGGATTAAATAAATTGTTTAATTGGTTACATCCTCGTCTTGAAGGAAGTACAATCTATGTAGGTGATCCTTACTGTCCTCCATCAGATAAAGAACATATATCATTAGAGGATCACCCCGATTCTGGATTTTACGTTCATAACCCAAGAGGGGGTATAGAAGGATTTTGTCAAAAATTGTGGACACTTATATCCATAAGTGCAATACATCTAGCAGCTGTTAGGATAGGTGTGAGGTAGGCGGAAATGGTGTATACAGGGGCGCCCCCGCCA

>HZ/HPIV3/165

TTCTGGCGATTCTCGCTCTCCCCCCCCGGGGGTCGAGATTGTTGAAGTATTTATAGCAGATAGTAAATTTGATCCTCATCAGATATTGGATTATGTAGAATCTGGGGATTGGTTAGATGATCCAGAATTTAATATTTCTTATAGTCTCAAAGAAAAGGAGATCAAACAAGAAGGTAGACTCTTTGCAAAAATGACATACAAAATGAGAGCTACACAGGTTTTATCAGAGACACTACTTGCAAACAATATAGGAAAATTCTTTCAAGAAAATGGGATGGTGAAAGGAGAGATTGAATTACTCAAGAGATTAACGACTATATCAATATCAGGAGTTCCACGATATAATGAAGTGTACAATAATTCTAAAAGCCATACAGATGATCTTAAGACTTATAATAAAATAAGTAATCTTAATTTATCCTCTAATCAGAAATCAAAGAAATTTGAATTCAAGTCAACAGATATTTACAATGATGGATATGAGACTGTGAGCTGTTTCCTAACAACAGATCTCAAAAAATATTGTCTTAACTGGAGATATGAATCAACAGCTCTATTTGGAGAAACTTGCAATCAAATATTTGGATTAAATAAATTGTTTAATTGGTTACATCCTCGTCTTGAAGGAAGTACAATCTATGTAGGTGATCCTTACTGTCCTCCATCAGATAAAGAGCATATATCATTAGAGGATCACCCTGATTCTGGATTTTACGTTCATAACCCAAGAGGGGGTATAGAAGGATTTTGTCAAAAATTGTGGACACTTATATCCATAAGTGCAATACATCTAGCAGCTGTTAGAATAGGTGTGAGGTGAGTTCAAAATGTGGTT

>HZ/HPIV3/293

TCTTCCCAAAAAATCGAGATAGTTGAAGTATTTATAGCAGATAGTAAATTTGATCCTCATCAGATATTGGATTATGTAGAATCTGGGGATTGGTTAGATGATCCAGAATTTAATATTTCTTATAGTCTCAAAGAAAAGGAGATCAAACAAGAAGGTAGACTCTTTGCAAAAATGACATACAAAATGAGAGCTACACAGGTTTTATCAGAGACACTACTTGCAAACAATATAGGAAAATTCTTTCAAGAAAATGGGATGGTGAAGGGAGAGATTGAATTACTTAAGAGATTAACGACTATATCAATATCAGGAGTTCCACGATATAATGAAGTGTACAATAATTCTAAAAGCCATACAGATGATCTTAAAACTTATAATAAAATAAGTAACCTTAATCTATCCTCTAATCAGAAATCAAAGAAATTTGAATTTAAGTCAACGGATATTTACAATGATGGATATGAGACTGTGAGCTGTTTCCTAACAACAGATCTCAAAAAATACTGTCTTAACTGGAGATATGAATCAACGGCTCTATTTGGAGAAACTTGCAATCAAATATTTGGATTAAATAAATTGTTTAATTGGTTACATCCTCGTCTTGAAGGAAGTACAATCTATGTAGGTGATCCTTACTGTCCTCCATCAGATAAAGAACATATATCATTAGAGGATCACCCTGATTCTGGATTCTACGTTCACAACCCAAGAGGGGGTATAGAAGGATTTTGTCAAAAATTATGGACACTTATATCCATAAGTGCAATACATCTAGCAGCTGTTAGGATAGGTGTGAGGAGCCCCAGGGGGGTGATACAAACGGGCCCCCTCTCCCCTTTCTTTCT

>HZ/HPIV3/440

CCCCCCCGAATGTGAAGTATTATAGCAGATAGTAAATTTGATCCTCATCAGATATTGGATTATGTAGAATCTGGGGATTGGTTAGATGATCCAGAATTTAATATTTCTTATAGTCTCAAAGAAAAGGAGATCAAACAAGAAGGTAGACTCTTTGCAAAAATGACATACAAAATGAGAGCTACACAGGTTTTATCAGAGACACTACTTGCAAACAATATAGGAAAATTCTTTCAAGAAAATGGGATGGTGAAGGGAGAGATTGAATTACTTAAGAGATTAACGACTATATCAATATCAGGAGTTCCACGATATAATGAAGTGTACAATAATTCTAAAAGCCATACAGATGATCTTAAAACTTATAATAAAATAAGTAATCTTAATTTATCTTCTAATCAGAAATCAAAGAAATTTGAATTCAAGTCAACGGATATTTACAATGATGGATATGAGACTGTAAGCTGTTTCCTAACAACAGATCTCAAAAAATATTGTCTTAACTGGAGATATGAATCAACAGCTCTATTTGGAGAAACTTGCAATCAAATATTTGGATTAAATAAATTGTTTAATTGGTTACATCCTCGTCTTGAAGGAAGTACAATCTATGTAGGTGATCCTTACTGTCCTCCATCAGATAAAGAACATATATCATTAGAGGATCACCCCGATTCTGGATTTTACGTTCATAACCCAAGAGGGGGTATAGAAGGATTTTGTCAAAAATTGTGGACACTTATATCCATAAGTGCAATACATCTAGCAGCTGTAGGAAGGGGAGGGGGGGGG

>HZ/HPIV3/458

TTTTCCCAAAAGGCACCGAAGATTAGTTGAAGTATTTATAGCAGATAGTAAATTTGATCCTCATCAGATATTGGATTATGTAGAATCTGGGGATTGGTTAGATGATCCAGAATTTAATATTTCTTATAGTCTCAAAGAAAAGGAGATCAAACAAGAAGGTAGACTCTTTGCAAAAATGACATACAAAATGAGAGCTACACAGGTTTTATCAGAGACACTACTTGCAAACAATATAGGAAAATTCTTTCAAGAAAATGGGATGGTGAAAGGAGAGATTGAATTACTTAAGAGATTAACGACTATATCAATATCAGGAGTTCCACGATATAATGAAGTGTACAATAATTCTAAAAGCCATACAGATGATCTTAAGACTTATAATAAAATAAGTAATCTTAATTTATCCTCTAATCAGAAATCAAAGAAATTTGAATTCAAGTCAACAGATATTTACAATGATGGATATGAGACTGTGAGCTGTTTCCTAACAACAGATCTCAAAAAATATTGTCTTAACTGGAGATATGAATCAACAGCTCTATTTGGAGAAACTTGCAATCAAATATTTGGATTAAATAAATTGTTTAATTGGTTACATCCTCGTCTTGAAGGAAGTACAATCTATGTAGGTGATCCTTACTGTCCCCCATCAGATAAAGAACATATATCATTAGAGGATCACCCTGATTCTGGATTTTACGTTCATAACCCAAGAGGGGGTATAGAAGGATTTTGTCAAAAATTGTGGACACTTATATCCATAAGTGCAATACATCTAGCAGCTGTTAGAATAGGTGTGAGGGCGGCCGGGAGGGGGT

>HZ/HPIV3/471

CCCCCCAACCGAATAGTGAAGTATTATAGCAGATAGTAAATTTGATCCTCATCAGATATTGGATTATGTAGAATCTGGGGATTGGTTAGATGATCCAGAATTTAATATTTCTTATAGTCTCAAAGAAAAGGAGATCAAACAAGAAGGTAGACTCTTTGCAAAAATGACATACAAAATGAGAGCTACACAGGTTTTATCAGAGACACTACTTGCAAACAATATAGGAAAATTCTTTCAAGAAAATGGGATGGTGAAAGGAGAGATTGAATTACTTAAGAGATTAACGACTATATCAATATCAGGAGTTCCACGATATAATGAAGTGTACAATAATTCTAAAAGCCATACAGATGATCTTAAGACTTATAATAAAATAAGTAATCTTAATTTATCCTCCAATCAGAAATCAAAGAAATTTGAATTCAAGTCAACAGATATTTACAATGATGGATATGAGACTGTGAGCTGTTTCCTAACAACAGATCTCAAAAAATATTGTCTTAACTGGAGATATGAATCAACAGCTCTATTTGGAGAAACTTGCAATCAAATATTTGGATTAAATAAATTGTTTAATTGGTTACATCCTCGTCTTGAAGGAAGTACAATCTATGTAGGTGATCCTTACTGTCCTCCATCAGATAAAGAACATATATCATTAGAGGATCACCCTGATTCTGGATTTTACGTTCATAACCCAAGAGGGGGTATAGAAGGATTTTGTCAAAAATTGTGGACACTTATATCCATAAGTGCAATACATCTAGCAGCTGTAGAATAGGGTAGCGGGGGGGGGGGGGTGGTAAAAAA

>HZ/HPIV3/492

CAAAACCGAATGTGAAGTATTATAGCAGATAGTAAATTTGATCCTCATCAGATATTGGATTATGTAGAGTCTGGGGATTGGTTAGATGATCCAGAATTTAATATTTCTTATAGTCTCAAAGAAAAGGAGATCAAACAAGAAGGTAGACTCTTTGCAAAAATGACATACAAAATGAGAGCTACACAGGTTTTATCAGAGACACTACTTGCAAACAATATAGGAAAATTCTTTCAAGAAAATGGGATGGTGAAAGGAGAGATTGAATTACTTAAGAGATTAACGACTATATCAATATCAGGAGTTCCACGATATAATGAAGTGTACAATAATTCTAAAAGCCATACAGATGATCTTAAGACTTATAATAAAATAAGTAATCTTAATTTATCCTCCAATCAGAAATCAAAGAAATTTGAATTCAAGTCAACAGATATTTACAATGATGGATATGAGACTGTGAGCTGTTTCCTAACAACAGATCTCAAAAAATATTGTCTTAACTGGAGATATGAATCAACAGCTCTATTTGGAGAAACTTGCAATCAAATATTTGGATTAAATAAATTGTTTAATTGGTTACATCCTCGTCTTGAAGGAAGTACAATCTATGTAGGTGATCCTTACTGTCCTCCATCAGATAAAGAACATATATCATTAGAGGATCACCCTGATTCTGGATTTTACGTTCATAACCCAAGAGGGGGTATAGAAGGATTTTGTCAAAAATTGTGGACACTTATATCCATAAGTGCAATACATCTAGCAGCTGTTAGAAAGGTGAGCGCGGGGGG

>HZ/HPIV3/503

CCCCCCGATTGTGAGTATTATAGCAGATAGTAAATTTGATCCTCATCAGATATTGGATTATGTAGAATCTGGGGATTGGTTAGATGATCCAGAATTTAATATTTCTTATAGTCTCAAAGAAAAGGAGATCAAACAAGAAGGTAGACTCTTTGCAAAAATGACATACAAAATGAGAGCTACACAGGTTTTATCAGAGACACTACTTGCAAACAATATAGGAAAATTCTTTCAAGAAAATGGGATGGTGAAGGGAGAGATTGAATTACTTAAGAGATTAACGACTATATCAATATCAGGAGTTCCACGATATAATGAAGTGTACAATAATTCTAAAAGCCATACAGATGATCTTAAGACTTATAATAAAATAAGTAATCTTAATTTATCTTCTAATCAGAAATCAAAGAAATTTGAATTCAAGTCAACAGATATTTACAATGATGGATATGAGACTGTGAGCTGTTTCCTAACAACAGATCTCAAAAAATATTGTCTTAACTGGAGATATGAATCAACAGCTCTATTTGGAGAAACTTGCAATCAAATATTTGGATTAAATAAATTGTTTAATTGGTTACATCCTCGTCTTGAAGGAAGTACAATCTATGTAGGTGATCCTTACTGTCCTCCATCAGATAAAGAACATATATCATTAGAGGATCACCCTGATTCTGGATTTTACGTTCATAACCCAAGAGGGGGTATAGAAGGATTTTGTCAAAAATTGTGGACACTTATATCCATAAGTGCAATACATCTAGCAGCTGTTAGATAGTGTAAGGGGGGGGGGGGGGGGGGGGAGGAAAAA

>HZ/HPIV3/510

CAAACACCCCGAGATAGTGAAGTATTATAGCAGATAGTAAATTTGATCCTCATCAGATATTGGATTATGTAGAATCTGGGGATTGGTTAGATGATCCAGAATTTAATATTTCTTATAGTCTCAAAGAAAAGGAGATCAAACAAGAAGGTAGACTCTTTGCAAAAATGACATACAAAATGAGAGCTACACAGGTTTTATCAGAGACACTACTTGCAAACAATATAGGAAAATTCTTTCAAGAAAATGGGATGGTGAAAGGAGAGATTGAATTACTTAAGAGATTAACGACTATATCAATATCAGGAGTTCCACGATATAATGAAGTGTACAATAATTCTAAAAGCCATACAGATGATCTTAAGACTTATAATAAAATAAGTAATCTTAATTTATCCTCCAATCAGAAATCAAAGAAATTTGAATTCAAGTCAACAGATATTTACAATGATGGATATGAGACTGTGAGCTGTTTCCTAACAACAGATCTCAAAAAATATTGTCTTAACTGGAGATATGAATCAACAGCTCTATTTGGAGAAACTTGCAATCAAATATTTGGATTAAATAAATTGTTTAATTGGTTACATCCTCGTCTTGAAGGAAGTACAATCTATGTAGGTGATCCTTACTGTCCTCCATCAGATAAAGAACATATATCATTAGAGGATCACCCTGATTCTGGATTTTACGTTCATAACCCAAGAGGGGGTATAGAAGGATTTTGTCAAAAATTGTGGACACTTATATCCATAAGTGCAATACATCTAGCAGCTGTTAAATAGGTGGAGTAGTGCCCGTTTG

>HZ/HPIV3/513

CCCCCCCCACGAGATTAGTGAAGTATTTATAGCAGATAGTAAATTTGATCCTCATCAGATATTAGATTATGTAGAATCTGGGGATTGGTTAGATGATCCAGAATTTAATATTTCTTATAGTCTCAAAGAAAAGGAGATCAAACAGGAAGGTAGACTCTTTGCAAAAATGACATACAAAATGAGAGCTACACAGGTTTTATCAGAGACACTACTTGCAAACAATATAGGAAAATTCTTTCAAGAAAATGGGATGGTGAAAGGAGAGATTGAATTACTTAAGAGATTAACGACTATATCAATATCAGGAGTTCCACGATATAATGAAGTGTACAATAATTCTAAAAGCCATACAGATGATCTTAAAACTTATAATAAAATAAGTAATCTTAATTTATCCTCTAATCAAAAATCAAAGAAATTTGAATTCAAGTCAACGGATATTTACAATGATGGATATGAGACTGTGAGCTGTTTCCTAACAACAGATCTCAAAAAATACTGTCTCAACTGGAGATATGAATCAACGGCTCTATTTGGAGAAACCTGCAATCAAATATTTGGATTAAATAAATTGTTTAATTGGTTACATCCTCGTCTTGAAGGAAGTACAATCTATGTAGGTGATCCTTACTGTCCTCCATCAGATAAGGAACATATATCATTAGAGGATCACCCTGATTCTGGATTTTACGTTCATAACCCAAGAGGGGGTATAGAAGGATTTTGTCAAAAATTGTGGACACTTATATCCATAAGTGCAATACATCTAGCAGCTGTTAGGATAGGTGTGAGGAGCCTTTTAGTGTGGTGAAAAAAAAGGCC

>HZ/HPIV3/529

AAACGGGGTAGAGATTAGTGAAGTATTTATAGCAGATAGTAAATTTGATCCTCATCAGATATTGGATTATGTAGAATCTGGGGATTGGTTAGATGATCCGGAATTTAATATTTCTTATAGTCTCAAAGAAAAGGAGATCAAACAAGAAGGTAGACTCTTTGCAAAAATGACATACAAAATGAGAGCTACACAGGTTTTATCAGAGACACTACTTGCAAACAATATAGGAAAATTCTTTCAAGAAAATGGGATGGTGAAAGGAGAGATTGAATTACTTAAGAGATTAACGACTATATCAATATCAGGAGTTCCACGATATAATGAAGTGTACAATAATTCTAAAAGCCATACAGATGATCTTAAGACTTATAATAAAATAAGTAATCTTAATTTATCCTCTAATCAGAAATCAAAGAAATTTGAATTCAAGTCAACAGATATTTACAATGATGGATATGAGACTGTGAGCTGTTTCCTAACAACAGATCTCAAAAAATATTGTCTTAACTGGAGATATGAATCAACAGCTCTATTTGGAGAAACTTGCAATCAAATATTTGGATTAAATAAATTGTTTAATTGGTTACATCCTCGTCTTGAAGGAAGTACAATCTATGTAGGTGATCCTTACTGTCCTCCATCAGATAAAGAACATATATCATTAGAGGATCACCCTGATTCTGGATTTTACGTTCATAACCCAAGAGGGGGTATAGAAGGATTTTGTCAAAAATTGTGGACACTTATATCCATAAGTGCAATACATCTAGCAGCTGTTAAATAGGTGGAGGAGGGGGGGGGGGGGGGGAAAAAAGGGGCGG

>HZ/HPIV3/531

CCCGCCCACACGAGATAGTTGAAGTATTTATAGCAGATAGTAAATTTGATCCTCATCAGATATTGGATTATGTAGAATCTGGGGATTGGTTAGATGATCCAGAATTTAATATTTCTTATAGTCTCAAAGAAAAGGAGATCAAACAAGAAGGTAGACTCTTTGCAAAAATGACATACAAAATGAGAGCTACACAGGTTTTATCAGAGACACTACTTGCAAACAATATAGGAAAATTCTTTCAAGAAAATGGGATGGTGAAGGGAGAGATTGAATTACTTAAGAGATTAACGACTATATCAATATCAGGAGTTCCACGATATAATGAAGTGTACAATAATTCTAAAAGCCATACAGATGATCTTAAAACTTATAATAAAATAAGTAATCTTAATTTATCTTCTAATCAGAAATCAAAGAAATTTGAATTCAAGTCAACGGATATTTACAATGATGGATATGAGACTGTAAGCTGTTTCCTAACCACAGATCTCAAAAAATATTGTCTTAACTGGAGATATGAATCAACAGCTCTATTTGGAGAAACTTGCAATCAAATATTTGGATTAAATAAATTGTTTAATTGGTTACATCCTCGTCTTGAAGGAAGTACAATCTATGTAGGTGATCCTTACTGTCCTCCATCAGATAAAGAACATATATCATTAGAGGATCACCCCGATTCTGGATTTTACGTTCATAACCCAAGAGGGGGTATAGAAGGATTTTGTCAAAAATTGTGGACACTTATATCCATAAGTGCAATACATCTAGCAGCTGTTAGGATAGGTGTGAGCGGGCTTTTG

>HZ/HPIV3/615

CCCCCAAACCGAATAGTGAAGTATTATAGCAGATAGTAAATTTGATCCTCATCAGATATTGGATTATGTAGAATCTGGGGATTGGTTAGATGATCCAGAATTTAATATTTCTTATAGTCTCAAAGAAAAGGAGATCAAACAAGAAGGTAGACTCTTTGCAAAAATGACATACAAAATGAGAGCTACACAGGTTTTATCAGAGACACTACTTGCAAACAATATAGGAAAATTCTTTCAAGAAAATGGGATGGTGAAGGGAGAGATTGAATTACTTAAGAGATTAACGACTATATCAATATCAGGAGTTCCACGATATAATGAAGTGTACAATAATTCTAAAAGCCATACAGATGATCTTAAAACTTATAATAAAATAAGTAACCTTAATCTATCCTCTAATCAGAAATCAAAGAAATTTGAATTTAAGTCAACGGATATTTACAATGATGGATATGAGACTGTGAGCTGTTTCCTAACAACAGATCTCAAAAAATACTGTCTTAACTGGAGATATGAATCAACGGCTCTATTTGGAGAAACTTGCAATCAAATATTTGGATTAAATAAATTGTTTAATTGGTTACATCCTCGTCTTGAAGGAAGTACAATCTATGTAGGTGATCCTTACTGTCCTCCATCAGATAAAGAACATATATCATTAGAGGATCACCCTGATTCTGGATTCTACGTTCACAACCCAAGAGGGGGTATAGAAGGATTTTGTCAAAAATTATGGACACTTATATCCATAAGTGCAATACATCTAGCAGCTGTAGGAAGGTGGAGGGGGGGGG

>HZ/HPIV3/757

ATTCAAATCCCCCCACCCCCCCCCCCCCCCCGCCGAGATAGTGAAGTATTTATAGCAGATAGTAAATTTGATCCTCATCAGATATTGGATTATGTAGAATCTGGGGATTGGTTAGATGATCCAGAATTTAATATTTCTTATAGTCTCAAAGAAAAGGAGATCAAACAAGAAGGTAGACTCTTTGCAAAAATGACATACAAAATGAGAGCTACACAGGTTTTATCAGAGACACTACTTGCAAACAATATAGGAAAATTCTTTCAAGAAAATGGGATGGTGAAGGGAGAGATTGAATTACTTAAGAGATTAACGACTATATCAATATCAGGAGTTCCACGATATAATGAAGTGTACAATAATTCTAAAAGCCATACAGATGATCTTAAAACTTATAATAAAATAAGTAATCTTAATTTATCTTCTAATCAGAAATCAAAGAAATTTGAATTCAAGTCAACGGATATTTACAATGATGGATATGAGACTGTAAGCTGTTTCCTAACAACAGATCTCAAAAAATATTGTCTTAACTGGAGATATGAATCAACAGCTCTATTTGGAGAAACTTGCAATCAAATATTTGGATTAAATAAATTGTTTAATTGGTTACATCCTCGTCTTGAAGGAAGTACAATCTATGTAGGTGATCCTTACTGTCCTCCATCAGATAAAGAACATATATCATTAGAGGATCACCCCGATTCTGGATTTTACGTTCATAACCCAAGAGGGGGTATAGAAGGATTTTGTCAAAAATTGTGGACACTTATATCCATAAGTGCAATACATCTAGCAGCTGTTAGGATAGGTGTGGCGGGGGG

>HZ/HPIV3/809

CCAAACCCCGAGTTAGTGAAGTATTTATAGCAGATAGTAAATTTGATCCTCATCAGATATTAGATTATGTAGAATCTGGGGATTGGTTAGATGATCCAGAATTTAATATTTCTTACAGTCTCAAAGAAAAGGAGATCAAACAAGAAGGTAGACTCTTTGCAAAAATGACATACAAAATGAGAGCTACACAGGTTTTATCAGAGACACTACTTGCAAACAATATAGGAAAATTCTTTCAAGAAAATGGGATGGTGAAGGGAGAGATTGAATTACTTAAGAGATTAACGACTATATCAATATCAGGAGTTCCACGATATAATGAAGTGTACAATAATTCTAAAAGCCATACGGATGATCTTAAAACTTATAATAAAATAAGTAATCTTAATTTATCCTCTAATCAAAAATCAAAGAAATTTGAATTCAAGTCAACGGATATTTACAATGATGGATATGAGACTGTGAGCTGTTTCCTAACAACAGATCTCAAAAAATACTGTCTTAACTGGAGATATGAATCAACGGCTCTATTTGGAGAAACTTGCAATCAAATATTTGGATTAAATAAATTGTTTAATTGGTTACATCCTCGTCTTGAAGGAAGTACAATCTATGTAGGTGATCCTTACTGTCCTCCATCAGATAAAGAACATATATCATTAGAGGATCACCCTGATTCTGGATTTTACGTTCATAACCCAAGAGGGGGTATAGAAGGATTTTGTCAAAAATTGTGGACACTTATATCCATAAGTGCAATACATCTAGCAGCTGTTAGGAAGGTGGAGCGGTTGGGGG

>HZ/HPIV3/811

CCCCCCCCCCGAATAGTGAAGTATTATAGCAGATAGTAAATTTGATCCTCATCAGATATTGGATTATGTAGAATCTGGGGATTGGTTAGATGATCCAGAATTTAATATTTCTTATAGTCTCAAAGAAAAGGAGATCAAACAAGAAGGTAGACTCTTTGCAAAAATGACATACAAAATGAGAGCTACACAGGTTTTATCAGAGACACTACTTGCAAACAATATAGGAAAATTCTTTCAAGAAAATGGGATGGTGAAAGGAGAGATTGAATTACTTAAGAGATTAACGACTATATCAATATCAGGAGTTCCACGATATAATGAAGTGTACAATAATTCTAAAAGCCATACAGATGATCTTAAGACTTATAATAAAATAAGTAATCTTAATTTATCCTCCAATCAGAAATCAAAGAAATTTGAATTCAAGTCAACAGATATTTACAATGATGGATATGAGACTGTGAGCTGTTTCCTAACAACAGATCTCAAAAAATATTGTCTTAACTGGAGATATGAATCAACAGCTCTATTTGGAGAAACTTGCAATCAAATATTTGGATTAAATAAATTGTTTAATTGGTTACATCCTCGTCTTGAAGGAAGTACAATCTATGTAGGTGATCCTTACTGTCCTCCATCAGATAAAGAACATATATCATTAGAGGATCACCCTGATTCTGGATTTTACGTTCATAACCCAAGAGGGGGTATAGAAGGATTTTGTCAAAAATTGTGGACACTTATATCCATAAGTGCAATACATCTAGCAGCTGTTAGAAAGGTGGAGAGGGGGGGGGG

>HZ/HPIV3/814

CCCCCCCCGAGTTGTGAAGTATTATAGCAGATAGTAAATTTGATCCTCATCAGATATTAGATTATGTAGAATCTGGGGATTGGTTAGATGATCCAGAATTTAATATTTCTTATAGTCTCAAAGAAAAGGAGATCAAACAGGAAGGTAGACTCTTTGCAAAAATGACATACAAAATGAGAGCTACACAGGTTTTATCAGAGACACTACTTGCAAACAATATAGGAAAATTCTTTCAAGAAAATGGGATGGTGAAAGGAGAGATTGAATTACTTAAGAGATTAACGACTATATCAATATCAGGAGTTCCACGATATAATGAAGTGTACAATAATTCTAAAAGCCATACAGATGATCTTAAAACTTATAATAAAATAAGTAATCTTAATTTATCCTCTAATCAAAAATCAAAGAAATTTGAATTCAAGTCAACGGATATTTACAATGATGGATATGAGACTGTGAGCTGTTTCCTAACAACAGATCTCAAAAAATACTGTCTCAACTGGAGATATGAATCAACGGCTCTATTTGGAGAAACCTGCAATCAAATATTTGGATTAAATAAATTGTTTAATTGGTTACATCCTCGTCTTGAAGGAAGTACAATCTATGTAGGTGATCCTTACTGTCCTCCATCAGATAAGGAACATATATCATTAGAGGATCACCCTGATTCTGGATTTTACGTTCATAACCCAAGAGGGGGTATAGAAGGATTTTGTCAAAAATTGTGGACACTTATATCCATAAGTGCAATACATCTAGCAGCTGTAGGAAGGTGAGCGAAGGGG

>HZ/HPIV3/820

CCCCCCCCCGAATTGTGAAGTATTTATAGCAGATAGTAAATTTGATCCTCATCAGATATTAGATTATGTAGAATCTGGGGATTGGTTAGATGATCCAGAATTTAATATTTCTTATAGTCTCAAAGAAAAAGAGATCAAACAGGAAGGTAGACTCTTTGCAAAAATGACATACAAAATGAGAGCTACACAGGTTTTATCAGAGACACTACTTGCAAACAATATAGGAAAATTCTTTCAAGAAAATGGGATGGTGAAGGGAGAGATTGAATTACTTAAGAGATTAACGACTATATCAATATCAGGAGTTCCACGATATAATGAAGTGTACAATAATTCTAAAAGCCATACAGATGATCTTAAAACTTATAATAAAATAAGTAATCTTAATTTATCCTCTAATCAAAAATCAAAGAAATTTGAATTCAAGTCAACGGATATTTACAATGATGGATATGAGACTGTGAGCTGTTTCCTAACAACAGATCTCAAAAAATACTGTCTCAACTGGAGATATGAATCAACGGCTCTATTTGGAGAAACTTGCAATCAAATATTTGGATTAAATAAATTGTTTAATTGGTTACATCCTCGTCTTGAAGGAAGTACGATCTATGTAGGTGATCCTTACTGTCCTCCATCAGATAAAGAACATATATCATTAGAGGATCACCCTGATTCTGGATTTTACGTTCATAACCCAAGAGGGGGTATAGAAGGATTTTGTCAAAAATTGTGGACACTTATATCCATAAGTGCAATACATCTAGCAGCTGTAGGATAGGTGGAGGGGGTGA

>HZ/HPIV3/821

CGAAAAGAACGAGATAGTGAAGTATTATAGCAGATAGTAAATTTGATCCTCATCAGATATTGGATTATGTAGAATCTGGGGATTGGTTAGATGATCCAGAATTTAATATTTCTTATAGTCTCAAAGAAAAGGAGATCAAACAAGAAGGTAGACTCTTTGCAAAAATGACATACAAAATGAGAGCTACACAGGTTTTATCAGAGACACTACTTGCAAACAATATAGGAAAATTCTTTCAAGAAAATGGGATGGTGAAAGGAGAGATTGAATTACTTAAGAGATTAACGACTATATCAATATCAGGAGTTCCACGATATAATGAAGTGTACAATAATTCTAAAAGCCATACAGATGATCTTAAGACTTATAATAAAATAAGTAATCTTAATTTATCCTCCAATCAGAAATCAAAGAAATTTGAATTCAAGTCAACAGATATTTACAATGATGGATATGAGACTGTGAGCTGTTTCCTAACAACAGATCTCAAAAAATATTGTCTTAACTGGAGATATGAATCAACAGCTCTATTTGGAGAAACTTGCAATCAAATATTTGGATTAAATAAATTGTTTAATTGGTTACATCCTCGTCTTGAAGGAAGTACAATCTATGTAGGTGATCCTTACTGTCCTCCATCAGATAAAGAACATATATCATTAGAGGATCACCCTGATTCTGGATTTTACGTTCATAACCCAAGAGGGGGTATAGAAGGATTTTGTCAAAAATTGTGGACACTTATATCCATAAGTGCAATACATCTAGCAGCTGTTAGAATAGGTGGAGCGGGGGGGGG

>HZ/HPIV3/822

CAACCCAGCGAGATAGTGAAGTATTATAGCAGATAGTAAATTTGATCCTCATCAGATATTGGATTATGTAGAATCTGGGGATTGGTTAGATGATCCAGAATTTAATATTTCTTATAGTCTCAAAGAAAAGGAGATCAAACAAGAAGGTAGACTCTTTGCAAAAATGACATACAAAATGAGAGCTACACAGGTTTTATCAGAGACACTACTTGCAAACAATATAGGAAAATTCTTTCAAGAAAATGGGATGGTGAAGGGAGAGATTGAATTACTTAAGAGATTAACGACTATATCAATATCAGGAGTTCCACGATATAATGAAGTGTACAATAATTCTAAAAGCCATACAGATGATCTTAAGACTTATAATAAAATAAGTAATCTTAATTTATCTTCTAATCAGAAATCAAAGAAATTTGAATTCAAGTCAACAGATATTTACAATGATGGATATGAGACTGTGAGCTGTTTCCTAACAACAGATCTCAAAAAATATTGTCTTAACTGGAGATATGAATCAACAGCTCTATTTGGAGAAACTTGCAATCAAATATTTGGATTAAATAAATTGTTTAATTGGTTACATCCTCGTCTTGAAGGAAGTACAATCTATGTAGGTGATCCTTACTGTCCTCCATCAGATAAAGAACATATATCATTAGAGGATCACCCTGATTCTGGATTTTACGTTCATAACCCAAGAGGGGGTATAGAAGGATTTTGTCAAAAATTGTGGACACTTATATCCATAAGTGCAATACATCTAGCAGCTGTAGAAAGGGAGGGGGGGGG

>HZ/HPIV3/823

CCCCCCCCGAGTAGTGAAGTATTATAGCAGATAGTAAATTTGATCCTCATCAGATATTGGATTATGTAGAATCTGGGGATTGGTTAGATGATCCAGAATTTAATATTTCTTATAGTCTCAAAGAAAAGGAGATCAAACAAGAAGGTAGACTCTTTGCAAAAATGACATACAAAATGAGAGCTACACAGGTTTTATCAGAGACACTACTTGCAAACAATATAGGAAAATTCTTTCAAGAAAATGGGATGGTGAAGGGAGAGATTGAATTACTTAAGAGATTAACGACTATATCAATATCAGGAGTTCCACGATATAATGAAGTGTACAATAATTCTAAAAGCCATACAGATGATCTTAAAACTTATAATAAAATAAGTAACCTTAATCTATCCTCTAATCAGAAATCAAAGAAATTTGAATTTAAGTCAACGGATATTTACAATGATGGATATGAGACTGTGAGCTGTTTCCTAACAACAGATCTCAAAAAATACTGTCTTAACTGGAGATATGAATCAACGGCTCTATTTGGAGAAACTTGCAATCAAATATTTGGATTAAATAAATTGTTTAATTGGTTACATCCTCGTCTTGAAGGAAGTACAATCTATGTAGGTGATCCTTACTGTCCTCCATCAGATAAAGAACATATATCATTAGAGGATCACCCTGATTCTGGATTCTACGTTCACAACCCAAGAGGGGGTATAGAAGGATTTTGTCAAAAATTATGGACACTTATATCCATAAGTGCAATACATCTAGCAGCTGTAGGAAGGGAGCGGATCGGG

>HZ/HPIV3/824

CAGCCGAATGTGAAGTATTATAGCAGATAGTAAATTTGATCCTCATCAGATATTAGATTATGTAGAATCTGGGGATTGGTTAGATGATCCAGAATTTAATATTTCTTATAGTCTCAAAGAAAAAGAGATCAAACAGGAAGGTAGACTCTTTGCAAAAATGACATACAAAATGAGAGCTACACAGGTTTTATCAGAGACACTACTTGCAAACAATATAGGAAAATTCTTTCAAGAAAATGGGATGGTGAAGGGAGAGATTGAATTACTTAAGAGATTAACGACTATATCAATATCAGGAGTTCCACGATACAATGAAGTGTACAATAATTCTAAAAGCCATACAGATGATCTTAAAACTTATAATAAAATAAGTAATCTTAATTTATCCTCTAATCAAAAATCAAAGAAATTTGAATTCAAGTCAACGGATATTTACAATGACGGATATGAGACTGTGAGCTGTTTCCTAACAACAGATCTCAAAAAATACTGTCTCAACTGGAGATATGAATCAACGGCTCTATTTGGAGAAACTTGCAATCAAATATTTGGATTAAATAAATTGTTTAATTGGTTACATCCTCGTCTTGAAGGAAGTACAATCTATGTAGGTGATCCTTACTGTCCTCCATCAGATAAAGAACATATATCATTAGAGGATCACCCTGATTCTGGATTTTACGTTCATAACCCAAGAGGGGGTATAGAAGGATTTTGTCAAAAATTGTGGACACTTATATCCATAAGTGCAATACATCTAGCAGCTGTTAGGAAGGTGGAGGCGGGGGGG

>HZ/HPIV3/831

CCCCCGCGAGTTGTGAAGTATTATAGCAGATAGTAAATTTGATCCTCATCAGATATTGGATTATGTAGAATCTGGGGATTGGTTAGATGATCCAGAATTTAATATTTCTTATAGTCTCAAAGAAAAGGAGATCAAACAAGAAGGTAGACTCTTTGCAAAAATGACATACAAAATGAGAGCTACACAGGTTTTATCAGAGACACTACTTGCAAACAATATAGGAAAATTCTTTCAAGAAAATGGGATGGTGAAAGGAGAGATTGAATTACTTAAGAGATTAACGACTATATCAATATCAGGAGTTCCACGATATAATGAAGTGTACAATAATTCTAAAAGCCATACAGATGATCTTAAGACTTATAATAAAATAAGTAATCTTAATTTATCCTCTAATCAGAAATCAAAGAAATTTGAATTCAAGTCAACAGATATTTACAATGATGGATATGAGACTGTGAGCTGTTTCCTAACAACAGATCTCAAAAAATATTGTCTTAACTGGAGATATGAATCAACAGCTCTATTTGGAGAAACTTGCAATCAAATATTTGGATTAAATAAATTGTTTAATTGGTTACATCCTCGTCTTGAAGGAAGTACAATCTATGTAGGTGATCCTTACTGTCCTCCATCAGATAAAGAACATATATCATTAGAGGATCACCCTGATTCTGGATTTTACGTTCATAACCCAAGAGGGGGCATAGAAGGATTTTGTCAAAAATTGTGGACACTTATATCCATAAGTGCAATACATCTAGCAGCTGTTAGAATAGGTGGAGGCGCCGGGGGG

>HZ/HPIV3/840

CTTTTCCCACGGGGACAGAAGATTAGTTGAAGTATTTATAGCAGATAGTAAATTTGATCCTCATCAGATATTAGATTATGTAGAATCTGGGGATTGGTTAGATGATCCAGAATTTAATATTTCTTATAGTCTCAAAGAAAAGGAGATCAAACAGGAAGGTAGACTCTTTGCAAAAATGACATACAAAATGAGAGCTACACAGGTTTTATCAGAGACACTACTTGCAAACAATATAGGAAAATTCTTTCAAGAAAATGGGATGGTGAAAGGAGAGATTGAATTACTTAAGAGATTAACGACTATATCAATATCAGGGGTTCCACGATATAATGAAGTGTACAATAATTCTAAAAGCCATACAGATGATCTTAAAACTTATAATAAAATAAGTAATCTTAATTTATCCTCTAATCAAAAATCAAAGAAATTTGAATTCAAGTCAACGGATATTTACAATGATGGATATGAGACTGTGAGCTGTTTCCTAACAACAGATCTCAAAAAATACTGTCTCAACTGGAGATATGAATCAACGGCTCTATTTGGAGAAACTTGCAATCAAATATTTGGATTAAATAAATTGTTTAATTGGTTACATCCTCGTCTTGAAGGAAGTACAATCTATGTAGGTGATCCTTACTGTCCTCCATCAGATAAGGAACATATATCATTAGAGGATCACCCTGATTCTGGATTTTACGTTCATAACCCAAGAGGGGGTATAGAAGGATTTTGTCAAAAATTGTGGACACTTATATCCATAAGTGCAATACATCTAGCAGCTGTTAGGATAGGTGTGAGGGGACGGCAAAAGGGTTAAAACTCTCCCCC

>HZ/HPIV3/852

AAATCCCCCTTTCCAAAAGGGATCCGAAGATTAGTTGAAGTATTTATAGCAGATAGTAAATTTGATCCTCATCAGATATTGGATTATGTAGAATCTGGGGATTGGTTAGATGATCCAGAATTTAATATTTCTTATAGTCTCAAAGAAAAGGAGATCAAACAAGAAGGTAGACTCTTTGCAAAAATGACATACAAAATGAGAGCTACACAGGTTTTATCAGAGACACTACTTGCAAACAATATAGGAAAATTCTTTCAAGAAAATGGGATGGTGAAAGGAGAGATTGAATTACTTAAGAGATTAACGACTATATCAATATCAGGAGTTCCACGATATAATGAAGTGTACAATAATTCTAAAAGCCATGCAGATGATCTTAAGACTTATAATAAAATAAGTAATCTTAATTTATCCTCTAATCAGAAATCAAAGAAATTTGAATTCAAGTCAACAGATATTTACAATGATGGATATGAGACTGTGAGCTGTTTCCTAACAACAGATCTCAAAAAATATTGTCTTAACTGGAGATATGAATCAACAGCTCTATTTGGAGAAACTTGCAATCAAATATTTGGATTAAATAAATTGTTTAATTGGTTACATCCTCGTCTTGAAGGAAGTACAATCTATGTAGGTGATCCTTACTGTCCTCCATCAGATAAAGAACATATATCATTAGAGGATCACCCTGATTCTGGATTTTACGTTCATAACCCAAGAGGGGGTATAGAAGGATTTTGTCAAAAATTGTGGACACTTATATCCATAAGTGCAATACATCTAGCAGCTGTTAGAATAGGTGTGAGGGGATGGCCAAATGGGTTACAAAACG

>HZ/HPIV3/854

AAAAATTCCCCCTCTCCCCAGGGGACCGAAGATTAGTTGAAGTATTTATAGCAGATAGTAAATTTGATCCTCATCAGATATTGGATTATGTAGAATCTGGGGATTGGTTAGATGATCCAGAATTTAATATTTCTTATAGTCTCAAAGAAAAGGAGATCAAACAAGAAGGTAGACTCTTTGCAAAAATGACATACAAAATGAGAGCTACACAGGTTTTATCAGAGACACTACTTGCAAACAATATAGGAAAATTCTTTCAAGAAAATGGGATGGTGAAAGGAGAGATTGAATTACTTAAGAGATTAACGACTATATCAATATCAGGAGTTCCACGATATAATGAAGTGTACAATAATTCTAAAAGCCATACAGATGATCTTAAGACTTATAATAAAATAAGTAATCTTAATTTATCCTCTAATCAGAAATCAAAGAAATTTGAATTCAAGTCAACAGATATTTACAATGATGGATATGAGACTGTGAGCTGTTTCCTAACAACAGATCTCAAAAAATATTGTCTTAACTGGAGATATGAATCAACAGCTCTATTTGGAGAAACTTGCAATCAAATATTTGGATTAAATAAATTGTTTAATTGGTTACATCCTCGTCTTGAAGGAAGTACAATCTATGTAGGTGATCCTTACTGTCCTCCATCAGATAAAGAACATATATCATTAGAGGATCACCCTGATTCTGGATTTTACGTTCATAACCCAAGAGGGGGTATAGAAGGATTTTGTCAAAAATTGTGGACACTTATATCCATAAGTGCAATACATCTAGCAGCTGTTAGAATAGGTGTGAGGGGCGGGCCAAGGGGGGTACAAACCCCTCCCCC

>HZ/HPIV3/860

CCCCCCCCGAATATGAAGTATTATAGCAGATAGTAAATTTGATCCTCATCAGATATTGGATTATGTAGAATCTGGGGATTGGTTAGATGATCCAGAATTTAATATTTCTTATAGTCTCAAAGAAAAGGAGATCAAACAAGAAGGTAGACTCTTTGCAAAAATGACATACAAAATGAGAGCTACACAGGTTTTATCAGAGACACTACTTGCAAACAATATAGGAAAATTCTTTCAAGAAAATGGGATGGTGAAGGGAGAGATTGAATTACTTAAGAGATTAACGACTATATCAATATCAGGAGTTCCACGATATAATGAAGTGTACAATAATTCTAAAAGCCATACAGATGATCTTAAAACTTATAATAAAATAAGTAATCTTAATTTATCTTCTAATCAGAAATCAAAGAAATTTGAATTCAAGTCAACGGATATTTACAATGATGGATATGAGACTGTAAGCTGTTTCCTAACAACAGATCTCAAAAAATATTGTCTTAACTGGAGATATGAATCAACAGCTCTATTTGGAGAAACTTGCAATCAAATATTTGGATTAAATAAATTGTTTAATTGGTTACATCCTCGTCTTGAAGGAAGTACAATCTATGTAGGTGATCCTTACTGTCCTCCATCAGATAAAGAACATATATCATTAGAGGATCACCCCGATTCTGGATTTTACGTTCATAACCCAAGAGGGGGTATAGAAGGATTTTGTCAAAAATTGTGGACACTTATATCCATAAGTGCAATACATCTAGCAGCTGTTAGGATAGGTGGAGCGGCCGGGG

>HZ/HPIV3/861

AGAAAGCCCTGAGATAGTGAAGTATTTATAGCAGATAGTAAATTTGATCCTCATCAGATATTGGATTATGTAGAATCTGGGGATTGGTTAGATGATCCAGAATTTAATATTTCTTATAGTCTCAAAGAAAAGGAGATCAAACAAGAAGGTAGACTCTTTGCAAAAATGACATACAAAATGAGAGCTACACAGGTTTTATCAGAGACACTACTTGCAAACAATATAGGAAAATTCTTTCAAGAAAATGGGATGGTGAAGGGAGAGATTGAATTACTTAAGAGATTAACGACTATATCAATATCAGGAGTTCCACGATATAATGAAGTGTACAATAATTCTAAAAGCCATACAGATGATCTTAAAACTTATAATAAAATAAGTAATCTTAATTTATCTTCTAATCAGAAATCAAAGAAATTTGAATTCAAGTCAACGGATATTTACAATGATGGATATGAGACTGTAAGCTGTTTCCTAACAACAGATCTCAAAAAATATTGTCTTAACTGGAGATATGAATCAACAGCTCTATTTGGAGAAACTTGCAATCAAATATTTGGATTAAATAAATTGTTTAATTGGTTACATCCTCGTCTTGAAGGAAGTACAATCTATGTAGGTGATCCTTACTGTCCTCCATCAGATAAAGAACATATATCATTAGAGGATCACCCCGATTCTGGATTTTACGTTCATAACCCAAGAGGGGGTATAGAAGGATTTTGTCAAAAATTGTGGACACTTATATCCATAAGTGCAATACATCTAGCAGCTGTAGGAAGGGTAGGGCCCC

>HZ/HPIV3/864

AAAAAAAAAACCGAGTTAGTGAAGTATTATAGCAGATAGTAAATTTGATCCTCATCAGATATTGGATTATGTAGAATCTGGGGATTGGTTAGATGATCCAGAATTTAATATTTCTTATAGTCTCAAAGAAAAGGAGATCAAACAAGAAGGTAGACTCTTTGCAAAAATGACATACAAAATGAGAGCTACACAGGTTTTATCAGAGACACTACTTGCAAACAATATAGGAAAATTCTTTCAAGAAAATGGGATGGTGAAGGGAGAGATTGAATTACTTAAGAGATTAACGACTATATCAATATCAGGAGTTCCACGATATAATGAAGTGTACAATAATTCTAAAAGCCATACAGATGATCTTAAAACTTATAATAAAATAAGTAATCTTAATTTATCTTCTAATCAGAAATCAAAGAAATTTGAATTCAAGTCAACGGATATTTACAATGATGGATATGAGACTGTAAGCTGTTTCCTAACAACAGATCTCAAAAAATATTGTCTTAACTGGAGATATGAATCAACAGCTCTATTTGGAGAAACTTGCAATCAAATATTTGGATTAAATAAATTGTTTAATTGGTTACATCCTCGTCTTGAAGGAAGTACAATCTATGTAGGTGATCCTTACTGTCCTCCATCAGATAAAGAACATATATCATTAGAGGATCACCCCGATTCTGGATTTTACGTTCATAACCCAAGAGGGGGTATAGAAGGATTTTGTCAAAAATTGTGGACACTTATATCCATAAGTGCAATACATCTAGCAGCTGTTAGGAAGGTGGAGAGCCCTGGAA

>HZ/HPIV3/866

CCCCCAGAAGGCACGAGATTAGTTGAAGTATTTATAGCAGATAGTAAATTTGATCCTCATCAGATATTGGATTATGTAGAATCTGGGGATTGGTTAGATGATCCAGAATTTAATATTTCTTATAGTCTCAAAGAAAAGGAGATCAAACAAGAAGGTAGACTCTTTGCAAAAATGACATACAAAATGAGAGCTACACAGGTTTTATCAGAGACACTACTTGCAAACAATATAGGAAAATTCTTTCAAGAAAATGGGATGGTGAAGGGAGAGATTGAATTACTTAAGAGATTAACGACTATATCAATATCAGGAGTTCCACGATATAATGAAGTGTACAATAATTCTAAAAGCCATACAGATGATCTTAAAACTTATAATAAAATAAGTAATCTTAATTTATCTTCTAATCAGAAATCAAAGAAATTTGAATTCAAGTCAACGGATATTTACAATGATGGATATGAGACTGTAAGCTGTTTCCTAACAACAGATCTCAAAAAATATTGTCTTAACTGGAGATATGAATCAACAGCTCTATTTGGAGAAACTTGCAATCAAATATTTGGATTAAATAAATTGTTTAATTGGTTACATCCTCGTCTTGAAGGAAGTACAATCTATGTAGGTGATCCTTACTGTCCTCCATCAGATAAAGAACATATATCATTAGAGGATCACCCCGATTCTGGATTTTACGTTCATAACCCAAGAGGGGGTATAGAAGGATTTTGTCAAAAATTGTGGACACTTATATCCATAAGTGCAATACATCTAGCAGCTGTTAGGATAGGTGTGAGGGCGGGCGAAGGGGGGTATAAACCCCCCCCCCCCTCCACCGTAGTCC

>HZ/HPIV3/872

CCCCCCCCGAATATTAAGTATTATAGCAGATAGTAAATTTGATCCTCATCAGATATTGGATTATGTAGAATCTGGGGATTGGTTAGATGATCCAGAATTTAATATTTCTTATAGTCTCAAAGAAAAGGAGATCAAACAAGAAGGTAGACTCTTTGCAAAAATGACATACAAAATGAGAGCTACACAGGTTTTATCAGAGACACTACTTGCAAACAATATAGGAAAATTCTTTCAAGAAAATGGGATGGTGAAGGGAGAGATTGAATTACTTAAGAGATTAACGACTATATCAATATCAGGAGTTCCACGATATAATGAAGTGTACAATAATTCTAAAAGCCATACAGATGATCTTAAAACTTATAATAAAATAAGTAATCTTAATTTATCTTCTAATCAGAAATCAAAGAAATTTGAATTCAAGTCAACGGATATTTACAATGATGGATATGAGACTGTAAGCTGTTTCCTAACAACAGATCTCAAAAAATATTGTCTTAACTGGAGATATGAATCAACAGCTCTATTTGGAGAAACTTGCAATCAAATATTTGGATTAAATAAATTGTTTAATTGGTTACATCCTCGTCTTGAAGGAAGTACAATCTATGTAGGTGATCCTTACTGTCCTCCATCAGATAAAGAACATATATCATTAGAGGATCACCCCGATTCTGGATTTTACGTTCATAACCCAAGAGGGGGTATAGAAGGATTTTGTCAAAAATTGTGGACACTTATATCCATAAGTGCAATACATCTAGCAGCTGTAGGATAGTGGAGGGGGGG

>HZ/HPIV3/901

CCCCCCGCGAGTTAGTGAAGTATTTATAGCAGATAGTAAATTTGATCCTCATCAGATATTGGATTATGTAGAATCTGGGGATTGGTTAGATGATCCAGAATTTAATATTTCTTATAGTCTCAAAGAAAAGGAGATCAAACAAGAAGGTAGACTCTTTGCAAAAATGACATACAAAATGAGAGCTACACAGGTTTTATCAGAGACACTACTTGCAAACAATATAGGAAAATTCTTTCAAGAAAATGGGATGGTGAAAGGAGAGATTGAATTACTTAAGAGATTAACGACTATATCAATATCAGGAGTTCCACGATATAATGAAGTGTACAATAATTCTAAAAGCCATACAGATGATCTTAAGACTTATAATAAAATAAGTAATCTTAATTTATCCTCTAATCAGAAATCAAAGAAATTTGAATTCAAGTCAACAGATATTTACAATGATGGATATGAGACTGTGAGCTGTTTCCTAACAACAGATCTCAAAAAATATTGTCTTAACTGGAGATATGAATCAACAGCTCTATTTGGAGAAACTTGCAATCAAATATTTGGATTAAATAAATTGTTTAATTGGTTACATCCTCGTCTTGAAGGAAGTACAATCTATGTAGGTGATCCTTACTGTCCCCCATCAGATAAAGAACATATATCATTAGAGGATCACCCTGATTCTGGATTTTACGTTCATAACCCAAGAGGGGGTATAGAAGGATTTTGTCAAAAATTGTGGACACTTATATCCATAAGTGCAATACATCTAGCAGCTGTTAGAATAGGTGGAGAGGGGGCCGGG

>HZ/HPIV3/902

CCCCGACGGGTACGAGATTAGTTGAAGTATTTATAGCAGATAGTAAATTTGATCCTCATCAGATATTAGATTATGTAGAATCTGGGGATTGGTTAGATGATCCAGAATTTAATATTTCTTATAGTCTCAAAGAAAAAGAGATCAAACAGGAAGGTAGACTCTTTGCAAAAATGACATACAAAATGAGAGCTACACAGGTTTTATCAGAGACACTACTTGCAAACAATATAGGAAAATTCTTTCAAGAAAATGGGATGGTGAAGGGAGAGATTGAATTACTTAAGAGATTAACGACTATATCAATATCAGGAGTTCCACGATACAATGAAGTGTACAATAATTCTAAAAGCCATACAGATGATCTTAAAACTTATAATAAAATAAGTAATCTTAATTTATCCTCTAATCAAAAATCAAAGAAATTTGAATTCAAGTCAACGGATATTTACAATGACGGATATGAGACTGTGAGCTGTTTCCTAACAACAGATCTCAAAAAATACTGTCTCAACTGGAGATATGAATCAACGGCTCTATTTGGAGAAACTTGCAATCAAATATTTGGATTAAATAAATTGTTTAATTGGTTACATCCTCGTCTTGAAGGAAGTACAATCTATGTAGGTGATCCTTACTGTCCTCCATCAGATAAAGAACATATATCATTAGAGGATCACCCTGATTCTGGATTTTACGTTCATAACCCAAGAGGGGGTATAGAAGGATTTTGTCAAAAATTGTGGACACTTATATCCATAAGTGCAATACATCTAGCAGCTGTTAGGATAGGTGTGAGGGGCGCCCCCCCAGTGGTTACAA

>HZ/HPIV3/906

CACCCCCCCGCACGGTAGAGATTAGTGAAGTATTTATAGCAGATAGTAAATTTGATCCTCATCAGATATTGGATTATGTAGAATCTGGGGATTGGTTAGATGATCCAGAATTTAATATTTCTTATAGTCTCAAAGAAAAGGAGATCAAACAAGAAGGTAGACTCTTTGCAAAAATGACATACAAAATGAGAGCTACACAGGTTTTATCAGAGACACTACTTGCAAACAATATAGGAAAATTCTTTCAAGAAAATGGGATGGTAAAGGGAGAGATTGAATTACTTAAGAGATTAACGACTATATCAATATCAGGAGTTCCACGATATAATGAAGTGTACAATAATTCTAAAAGCCATACAGATGATCTTAAAACTTATAATAAAATAAGTAATCTTAATTTATCTTCTAATCAGAAATCAAAGAAATTTGAATTCAAGTCAACGGATATTTACAATGATGGATATGAGACTGTAAGCTGTTTCCTAACAACAGATCTCAAAAAATATTGTCTTAACTGGAGATATGAATCAACAGCTCTATTTGGAGAAACTTGCAATCAAATATTTGGATTAAATAAATTGTTTAATTGGTTACATCCTCGTCTTGAAGGAAGTACAATCTATGTAGGTGATCCTTACTGTCCTCCATCAGATAAAGAACATATATCATTAGAGGATCACCCCGATTCTGGATTTTACGTTCATAACCCAAGAGGGGGTATAGAAGGATTTTGTCAAAAATTGTGGACACTTATATCCATAAGTGCAATACATCTAGCAGCTGTTAGGATAGGTGTGAGGTGCGCCTCAAAAGTGGTATAATGGGG

>HZ/HPIV3/1063

AATTAAAAATCCCGCACTCACCCCCCCCGGAGCCGAGATTAGTGAAGTATTTATAGCAGATAGTAAATTTGATCCTCATCAGATATTAGATTATGTAGAATCTGGGGATTGGTTAGATGATCCAGAATTTAATATTTCTTATAGTCTCAAAGAAAAAGAGATCAAACAGGAAGGTAGACTCTTTGCAAAAATGACATACAAAATGAGAGCTACACAGGTTTTATCAGAGACACTACTTGCAAACAATATAGGAAAATTCTTTCAAGAAAATGGGATGGTGAAGGGAGAGATTGAATTACTTAAGAGATTAACGACTATATCAATATCAGGAGTTCCACGATACAATGAAGTGTACAATAATTCTAAAAGCCATACAGATGATCTTAAAACTTATAATAAAATAAGTAATCTTAATTTATCCTCTAATCAAAAATCAAAGAAATTTGAATTCAAGTCAACGGATATTTACAATGACGGATATGAGACTGTGAGCTGTTTCCTAACAACAGATCTCAAAAAATACTGTCTCAACTGGAGATATGAATCAACGGCTCTATTTGGAGAAACTTGCAATCAAATATTTGGATTAAATAAATTGTTTAATTGGTTACATCCTCGTCTTGAAGGAAGTACAATCTATGTAGGTGATCCTTACTGTCCTCCATCAGATAAAGAACATATATCATTAGAGGATCACCCTGATTCTGGATTTTACGTTCATAACCCAAGAGGGGGTATAGAAGGATTTTGTCAAAAATTGTGGACACTTATATCCATAAGTGCAATACATCTAGCAGCTGTTAGGATAGGTGGAGTGGCCGGGGGGA

>HZ/HPIV3/1107

AATCTCCAAATCGCGCTCTCTCCCCCCCGAGGTAGAGATTAGTGAAGTATTTATAGCAGATAGTAAATTTGATCCTCATCAGATATTGGATTATGTAGAATCTGGGGATTGGTTAGATGATCCAGAATTTAATATTTCTTATAGTCTCAAAGAAAAGGAGATCAAACAAGAAGGTAGACTCTTTGCAAAAATGACATACAAAATGAGAGCTACACAGGTTTTATCAGAGACACTACTTGCAAACAATATAGGAAAATTCTTTCAAGAAAATGGGATGGTGAAAGGAGAGATTGAATTACTTAAGAGATTAACGACTATATCAATATCAGGAGTTCCACGATATAATGAAGTGTACAATAATTCTAAAAGCCATACAGATGATCTTAAGACTTATAATAAAATAAGTAATCTTAATTTATCCTCTAATCAGAAATCAAAGAAATTTGAATTCAAGTCAACAGATATTTACAATGATGGATATGAGACTGTGAGCTGTTTCCTAACAACAGATCTCAAAAAATATTGTCTTAACTGGAGATATGAATCAACAGCTCTATTTGGAGAAACTTGCAATCAAATATTTGGATTAAATAAATTGTTTAATTGGTTACATCCTCGTCTTGAAGGAAGTACAATCTATGTAGGTGATCCTTACTGTCCCCCATCAGATAAAGAACATATATCATTAGAGGATCACCCTGATTCTGGATTTTACGTTCATAACCCAAGAGGGGGTATAGAAGGATTTTGTCAAAAATTGTGGACACTTATATCCATAAGTGCAATACATCTAGCAGCTGTTAGAATAGGTGTGAGGGACGCCCGCGAAGAGTGTATACATTCCTTTCCTCCTCCTCCACATTGGCAACGTCTCATCAACAAGTT

>HZ/HPIV3/1144

CACTATATTATTTCTCTCTCTCTTCTCCATTCTGGTAGAGATTAGTGAAGTATTTATAGCAGATAGTAAATTTGATCCTCATCAGATATTAGATTATGTAGAATCTGGGGATTGGTTAGATGATCCAGAATTTAATATTTCTTACAGTCTCAAAGAAAAGGAGATCAAACAAGAAGGTAGACTCTTTGCAAAAATGACATACAAAATGAGAGCTACACAGGTTTTATCAGAGACACTACTTGCAAACAATATAGGAAAATTCTTTCAAGAAAATGGGATGGTGAAGGGAGAGATTGAATTACTTAAGAGATTAACGACTATATCAATATCAGGAGTTCCACGATATAATGAAGTGTACAATAATTCTAAAAGCCATACGGATGATCTTAAAACTTATAATAAAATAAGTAATCTTAATTTATCCTCTAATCAAAAATCAAAGAAATTTGAATTCAAGTCAACGGATATTTACAATGATGGATATGAGACTGTGAGCTGTTTCCTAACAACAGATCTCAAAAAATACTGTCTTAACTGGAGATATGAATCAACGGCTCTATTTGGAGAAACTTGCAATCAAATATTTGGATTAAATAAATTGTTTAATTGGTTACATCCTCGTCTTGAAGGAAGTACAATCTATGTAGGTGATCCTTACTGTCCTCCATCAGATAAAGAACATATATCATTAGAGGATCACCCTGATTCTGGATTTTACGTTCATAACCCAAGAGGGGGTATAGAAGGATTTTGTCAAAAATTGTGGACACTTATATCCATAAGTGCAATACATCTAGCAGCTGTTAGGATAGGTGGAGGGCGCCGGAAGG

>HZ/HPIV3/1167

ATAATAATTACCGCACTCTCTCCCCCCAGCAGTAGAGATTAGTGAAGTATTTATAGCAGATAGTAAATTTGATCCTCATCAGATATTAGATTATGTAGAATCTGGGGATTGGTTAGATGATCCAGAATTTAATATTTCTTATAGTCTCAAAGAAAAAGAGATCAAACAGGAAGGTAGACTCTTTGCAAAAATGACATACAAAATGAGAGCTACACAGGTTTTATCAGAGACACTACTTGCAAACAATATAGGAAAATTCTTTCAAGAAAATGGGATGGTGAAGGGAGAGATTGAATTACTTAAGAGATTAACGACTATATCAATATCAGGAGTTCCACGATACAATGAAGTGTACAATAATTCTAAAAGCCATACAGATGATCTTAAAACTTATAATAAAATAAGTAATCTTAATTTATCCTCTAATCAAAAATCAAAGAAATTTGAATTCAAGTCAACGGATATTTACAATGACGGATATGAGACTGTGAGCTGTTTCCTAACAACAGATCTCAAAAAATACTGTCTCAACTGGAGATATGAATCAACGGCTCTATTTGGAGAAACTTGCAATCAAATATTTGGATTAAATAAATTGTTTAATTGGTTACATCCTCGTCTTGAAGGAAGTACAATCTATGTAGGTGATCCTTACTGTCCTCCATCAGATAAAGAACATATATCATTAGAGGATCACCCTGATTCTGGATTTTACGTTCATAACCCAAGAGGGGGTATAGAAGGATTTTGTCAAAAATTGTGGACACTTATATCCATAAGTGCAATACATCTAGCAGCTGTTAGGATAGGTGTGAGGGGCGCCCCAAAGGGTGTCACAAAAAGGCTC

>HZ/HPIV3/1182

GCCTTCCCCATGGGGACACGAAGATTAGTTGAAGTATTTATAGCAGATAGTAAATTTGATCCTCATCAGATATTGGATTATGTAGAATCTGGGGATTGGTTAGATGATCCGGAATTTAATATTTCTTATAGTCTCAAAGAAAAGGAGATCAAACAAGAAGGTAGACTCTTTGCAAAAATGACATACAAAATGAGAGCTACACAGGTTTTATCAGAGACACTACTTGCAAACAATATAGGAAAATTCTTTCAAGAAAATGGGATGGTGAAAGGAGAGATTGAATTACTTAAGAGATTAACGACTATATCAATATCAGGAGTTCCACGATATAATGAAGTGTACAATAATTCTAAAAGCCATACAGATGATCTTAAGACTTATAATAAAATAAGTAATCTTAATTTATCCTCTAATCAGAAATCAAAGAAATTTGAATTCAAGTCAACAGATATTTACAATGATGGATATGAGACTGTGAGCTGTTTCCTAACAACAGATCTCAAAAAATATTGTCTTAACTGGAGATATGAATCAACAGCTCTATTTGGAGAAACTTGCAATCAAATATTTGGATTAAATAAATTGTTTAATTGGTTACATCCTCGTCTTGAAGGAAGTACAATCTATGTAGGTGATCCTTACTGTCCTCCATCAGATAAAGAACATATATCATTAGAGGATCACCCTGATTCTGGATTTTACGTTCATAACCCAAGAGGGGGTATAGAAGGATTTTGTCAAAAATTGTGGACACTTATATCCATAAGTGCAATACATCTAGCAGCTGTTAGAATAGGTGTGAGGGGATGGGAAAAGGGTTAAACC

>HZ/HPIV3/1208

ACCCCAAAAAAACACAGAGATTAGTGAAGTATTTATAGCAGATAGTAAATTTGATCCTCATCAGATATTGGATTATGTAGAATCTGGGGATTGGTTAGATGATCCAGAATTTAATATTTCTTATAGTCTCAAAGAAAAGGAGATCAAACAAGAAGGTAGACTCTTTGCAAAAATGACATACAAAATGAGAGCTACACAGGTTTTATCAGAGACACTACTTGCAAACAATATAGGAAAATTCTTTCAAGAAAATGGGATGGTGAAGGGAGAGATTGAATTACTTAAGAGATTAACGACTATATCAATATCAGGAGTTCCACGATATAATGAAGTGTACAATAATTCTAAAAGCCATACAGATGATCTTAAGACTTATAATAAAATAAGTAATCTTAATTTATCTTCTAATCAGAAATCAAAGAAATTTGAATTCAAGTCAACAGATATTTACAATGATGGATATGAGACTGTGAGCTGTTTCCTAACAACAGATCTCAAAAAATATTGTCTTAACTGGAGATATGAATCAACAGCTCTATTTGGAGAAACTTGCAATCAAATATTTGGATTAAATAAATTGTTTAATTGGTTACATCCTCGTCTTGAAGGAAGTACAATCTATGTAGGTGATCCTTACTGTCCTCCATCAGATAAAGAACATATATCATTAGAGGATCACCCTGATTCTGGATTTTACGTTCATAACCCAAGAGGGGGTATAGAAGGATTTTGTCAAAAATTGTGGACACTTATATCCATAAGTGCAATACATCTAGCAGCTGTTAGAATAGGTGGAGGGCGCCCCGGGGAAAATGAAAAAA

>HZ/HPIV3/1210

CCCCCAAAGAGACCGAGATTAGTGAAGTATTTATAGCAGATAGTAAATTTGATCCTCATCAGATATTGGATTATGTAGAATCTGGGGATTGGTTAGATGATCCAGAATTTAATATTTCTTATAGTCTCAAAGAAAAGGAGATCAAACAAGAAGGTAGACTCTTTGCAAAAATGACATACAAAATGAGAGCTACACAGGTTTTATCAGAGACACTACTTGCAAACAATATAGGAAAATTCTTTCAAGAAAATGGGATGGTGAAGGGAGAGATTGAATTACTTAAGAGATTAACGACTATATCAATATCAGGAGTTCCACGATATAATGAAGTGTACAATAATTCTAAAAGCCATACAGATGATCTTAAAACTTATAATAAAATAAGTAATCTTAATTTATCTTCTAATCAGAAATCAAAGAAATTTGAATTCAAGTCAACGGATATTTACAATGATGGATATGAGACTGTAAGCTGTTTCCTAACAACAGATCTCAAAAAATATTGTCTTAACTGGAGATATGAATCAACAGCTCTATTTGGAGAAACTTGCAATCAAATATTTGGATTAAATAAATTGTTTAATTGGTTACATCCTCGTCTTGAAGGAAGTACAATCTATGTAGGTGATCCTTACTGTCCTCCATCAGATAAAGAACATATATCATTAGAGGATCACCCCGATTCTGGATTTTACGTTCATAACCCAAGAGGGGGTATAGAAGGATTTTGTCAAAAATTGTGGACACTTATATCCATAAGTGCAATACATCTAGCAGCTGTTAGGATAGGTGTGAGGTGCGTTTTAAAAAGTGGTAT

>HZ/HPIV3/1212

CCAAAGAACGCACGAGATTAGTTGAAGTATTTATAGCAGATAGTAAATTTGATCCTCATCAGATATTGGATTATGTAGAATCTGGGGATTGGTTAGATGATCCAGAATTTAATATTTCTTATAGTCTCAAAGAAAAGGAGATCAAACAAGAAGGTAGACTCTTTGCAAAAATGACATACAAAATGAGAGCTACACAGGTTTTATCAGAGACACTACTTGCAAACAATATAGGAAAATTCTTTCAAGAAAATGGGATGGTGAAAGGAGAGATTGAATTACTTAAGAGATTAACGACTATATCAATATCAGGAGTTCCACGATATAATGAAGTGTACAATAATTCTAAAAGCCATACAGATGATCTTAAGACTTATAATAAAATAAGTAATCTTAATTTATCCTCTAATCAGAAATCAAAGAAATTTGAATTCAAGTCAACAGATATTTACAATGATGGATATGAGACTGTGAGCTGTTTCCTAACAACAGATCTCAAAAAATATTGTCTTAACTGGAGATATGAATCAACAGCTCTATTTGGAGAAACTTGCAATCAAATATTTGGATTAAATAAATTGTTTAATTGGTTACATCCTCGTCTTGAAGGAAGTACAATCTATGTAGGTGATCCTTACTGTCCTCCATCAGATAAAGAACATATATCATTAGAGGATCACCCTGATTCTGGATTTTACGTTCATAACCCAAGAGGGGGTATAGAAGGATTTTGTCAAAAATTGTGGACACTTATATCCATAAGTGCAATACATCTAGCAGCTGTTAGAATAGGTGTGAGGGCGCCCCAAGAGTGGTAAAAA

>HZ/HPIV3/1222

TTCTCCCCAGGGAGACAGAAGATTAGTTGAAGTATTTATAGCAGATAGTAAATTTGATCCTCATCAGATATTGGATTATGTAGAATCTGGGGATTGGTTAGATGATCCAGAATTTAATATTTCTTATAGTCTCAAAGAAAAGGAGATCAAACAAGAAGGTAGACTCTTTGCAAAAATGACATACAAAATGAGAGCTACACAGGTTTTATCAGAGACACTACTTGCAAACAATATAGGAAAATTCTTTCAAGAAAATGGGATGGTGAAGGGAGAGATTGAATTACTTAAGAGATTAACGACTATATCAATATCAGGAGTTCCACGATATAATGAAGTGTACAATAATTCTAAAAGCCATACAGATGATCTTAAAACTTATAATAAAATAAGTAACCTTAATCTATCCTCTAATCAGAAATCAAAGAAATTTGAATTTAAGTCAACGGATATTTACAATGATGGATATGAGACTGTGAGCTGTTTCCTAACAACAGATCTCAAAAAATACTGTCTTAACTGGAGATATGAATCAACGGCTCTATTTGGAGAAACTTGCAATCAAATATTTGGATTAAATAAATTGTTTAATTGGTTACATCCTCGTCTTGAAGGAAGTACAATCTATGTAGGTGATCCTTACTGTCCTCCATCAGATAAAGAACATATATCATTAGAGGATCACCCTGATTCTGGATTCTACGTTCACAACCCAAGAGGGGGTATAGAAGGATTTTGTCAAAAATTATGGACACTTATATCCATAAGTGCAATACATCTAGCAGCTGTTAGGATAGGTGTGAGGGGACGGGCAAAAGGGGTAAAATTTCCCCCC

>HZ/HPIV3/1236

TTTTAAGAAGAGAAGTGGAATTTCCGATTTTATTTGTAAAGATAGTCCACTCTCGAGGGGGGATCAGAAGATTAGTTGAAGTATTTATAGCAGATAGTAAATTTGATCCTCATCAGATATTAGATTATGTAGAATCTGGGGATTGGTTAGATGATCCAGAATTTAATATTTCTTATAGTCTCAAAGAAAAAGAGATCAAACAGGAAGGTAGACTCTTTGCAAAAATGACATACAAAATGAGAGCTACACAGGTTTTATCAGAGACACTACTTGCAAACAATATAGGAAAATTCTTTCAAGAAAATGGGATGGTGAAGGGAGAGATTGAATTACTTAAGAGATTAACGACTATATCAATATCAGGAGTTCCACGATACAATGAAGTGTACAATAATTCTAAAAGCCATACAGATGATCTTAAAACTTATAATAAAATAAGTAATCTTAATTTATCCTCTAATCAAAAATCAAAGAAATTTGAATTCAAGTCAACGGATATTTACAATGACGGATATGAGACTGTGAGCTGTTTCCTAACAACAGATCTCAAAAAATACTGTCTCAACTGGAGATATGAATCAACGGCTCTATTTGGAGAAACTTGCAATCAAATATTTGGATTAAATAAATTGTTTAATTGGTTACATCCTCGTCTTGAAGGAAGTACAATCTATGTAGGTGATCCTTACTGTCCTCCATCAGATAAAGAACATATATCATTAGAGGATCACCCTGATTCTGGATTTTACGTTCATAACCCAAGAGGGGGTATAGAAGGATTTTGTCAAAAATTGTGGACACTTATATCCATAAGTGCAATACATCTAGCAGCTGTTAGGATAGGTGTGAGGTGATGGCCAAAAGGGTTAAAAAGGAAGCCTG

>HZ/HPIV3/1311

AACAAAAAAGAACAGAAGATTAGTTGAAGTATTTATAGCAGATAGTAAATTTGATCCTCATCAGATATTGGATTATGTAGAATCTGGGGATTGGTTAGATGATCCAGAATTTAATATTTCTTATAGTCTCAAAGAAAAGGAGATCAAACAAGAAGGTAGACTCTTTGCAAAAATGACATACAAAATGAGAGCTACACAGGTTTTATCAGAGACACTACTTGCAAACAATATAGGAAAATTCTTTCAAGAAAATGGGATGGTGAAGGGAGAGATTGAATTACTTAAGAGATTAACGACTATATCAATATCAGGAGTTCCACGATATAATGAAGTGTACAATAATTCTAAAAGCCATACAGATGATCTTAAAACTTATAATAAAATAAGTAATCTTAATTTATCTTCTAATCAGAAATCAAAGAAATTTGAATTCAAGTCAACGGATATTTACAATGATGGATATGAGACTGTAAGCTGTTTCCTAACAACAGATCTCAAAAAATATTGTCTTAACTGGAGATATGAATCAACAGCTCTATTTGGAGAAACTTGCAATCAAATATTTGGATTAAATAAATTGTTTAATTGGTTACATCCTCGTCTTGAAGGAAGTACAATCTATGTAGGTGATCCTTACTGTCCTCCATCAGATAAAGAACATATATCATTAGAGGATCACCCCGATTCTGGATTTTACGTTCATAACCCAAGAGGGGGTATAGAAGGATTTTGTCAAAAATTGTGGACACTTATATCCATAAGTGCAATACATCTAGCAGCTGTTAGGATAGGTGTGAGGGGACGGCCAAAGTGGTTACAAGTGGCCATCCA

>HZ/HPIV3/1397

CTCTCACCACAGGGTAGAGATTAGTTGAAGTATTTATAGCAGATAGTAAATTTGATCCTCATCAGATATTGGATTATGTAGAATCTGGGGATTGGTTAGATGATCCAGAATTTAATATTTCTTATAGTCTCAAAGAAAAGGAGATCAAACAAGAAGGTAGACTCTTTGCAAAAATGACATACAAAATGAGAGCTACACAGGTTTTATCAGAGACACTACTTGCAAACAATATAGGAAAATTCTTTCAAGAAAATGGGATGGTGAAGGGAGAGATTGAATTACTTAAGAGATTAACGACTATATCAATATCAGGAGTTCCACGATATAATGAAGTGTACAATAATTCTAAAAGCCATACAGATGATCTTAAGACTTATAATAAAATAAGTAATCTTAATTTATCTTCTAATCAGAAATCAAAGAAATTTGAATTCAAGTCAACAGATATTTACAATGATGGATATGAGACTGTGAGCTGTTTCCTAACAACAGATCTCAAAAAATATTGTCTTAACTGGAGATATGAATCAACAGCTCTATTTGGAGAAACTTGCAATCAAATATTTGGATTAAATAAATTGTTTAATTGGTTACATCCTCGTCTTGAAGGAAGTACAATCTATGTAGGTGATCCTTACTGTCCTCCATCAGATAAAGAACATATATCATTAGAGGATCACCCTGATTCTGGATTTTACGTTCATAACCCAAGAGGGGGTATAGAAGGATTTTGTCAAAAATTGTGGACACTTATATCCATAAGTGCAATACATCTAGCAGCTGTTAAATAGGTGTGAGGGAGCCCAAAAGGGTGGATACAAAAAACCCCCCCTCACC

>HZ/HPIV3/1400

CCCCCAAGCGGGGCAGAAGATTAGTTGAAGTATTTATAGCAGATAGTAAATTTGATCCTCATCAGATATTAGATTATGTAGAATCTGGGGATTGGTTAGATGATCCAGAATTTAATATTTCTTATAGTCTCAAAGAAAAAGAGATCAAACAGGAAGGTAGACTCTTTGCAAAAATGACATACAAAATGAGAGCTACACAGGTTTTATCAGAGACACTACTTGCAAACAATATAGGAAAATTCTTTCAAGAAAATGGGATGGTGAAGGGAGAGATTGAATTACTTAAGAGATTAACGACTATATCAATATCAGGAGTTCCACGATACAATGAAGTGTACAATAATTCTAAAAGCCATACAGATGATCTTAAAACTTATAATAAAATAAGTAATCTTAATTTATCCTCTAATCAAAAATCAAAGAAATTTGAATTCAAGTCAACGGATATTTACAATGACGGATATGAGACTGTGAGCTGTTTCCTAACAACAGATCTCAAAAAATACTGTCTCAACTGGAGATATGAATCAACGGCTCTATTTGGAGAAACTTGCAATCAAATATTTGGATTAAATAAATTGTTTAATTGGTTACATCCTCGTCTTGAAGGAAGTACAATCTATGTAGGTGATCCTTACTGTCCTCCATCAGATAAAGAACATATATCATTAGAGGATCACCCTGATTCTGGATTTTACGTTCATAACCCAAGAGGGGGTATAGAAGGATTTTGTCAAAAATTGTGGACACTTATATCCATAAGTGCAATACATCTAGCAGCTGTTAGGATAGGTGTGAGGTGACGTCCAAAAGTGGTTACAAAGGAATCCCCCTACAA

>HZ/HPIV3/1407

CCTCCCCCCGGGGCACAGAAGATTAGTTGAAGTATTTATAGCAGATAGTAAATTTGATCCTCATCAGATATTGGATTATGTAGAATCTGGAGATTGGTTAGATGATCCAGAATTTAATATTTCTTATAGTCTCAAAGAAAAGGAGATCAAACAAGAAGGTAGACTCTTTGCAAAAATGACATACAAAATGAGAGCTACACAGGTTTTATCAGAGACACTACTTGCAAACAATATAGGAAAATTCTTTCAAGAAAATGGGATGGTGAAGGGAGAGATTGAATTACTTAAGAGATTAACGACTATATCAATATCAGGAGTTCCACGATATAATGAAGTGTACAATAATTCTAAAAGCCATACAGATGATCTTAAGACTTATAATAAAATAAGTAATCTTAATTTATCTTCTAATCAGAAATCAAAGAAATTTGAATTCAAGTCAACAGATATTTACAATGATGGATATGAGACTGTGAGCTGTTTCCTAACAACAGATCTCAAAAAATATTGTCTTAACTGGAGATATGAATCAACAGCTCTATTTGGAGAAACTTGCAATCAAATATTTGGATTAAATAAATTGTTTAATTGGTTACATCCTCGTCTTGAAGGAAGTACAATCTATGTAGGTGATCCTTACTGTCCTCCATCAGATAAAGAACATATATCATTAGAGGATCACCCTGATTCTGGATTTTACGTTCATAACCCAAGAGGGGGTATAGAAGGATTTTGTCAAAAATTGTGGACACTTATATCCATAAGTGCAATACATCTAGCAGCTGTTAGAATAGGTGTGAGGTGCGGGCCACGGGGGGCACAAAAA
